# Mock community taxonomic classification performance of publicly available shotgun metagenomics pipelines

**DOI:** 10.1038/s41597-023-02877-7

**Published:** 2024-01-17

**Authors:** E. Michael Valencia, Katherine A. Maki, Jennifer N. Dootz, Jennifer J. Barb

**Affiliations:** 1 Translational Biobehavioral and Health Disparities Branch, National Institutes of Health Clinical Center, Bethesda, MD 20814 USA; 2 Biosystems and Biomaterials Division, National Institute of Standards and Technology, Gaithersburg, MD 20899 USA

**Keywords:** Classification and taxonomy, Metagenomics

## Abstract

Shotgun metagenomic sequencing comprehensively samples the DNA of a microbial sample. Choosing the best bioinformatics processing package can be daunting due to the wide variety of tools available. Here, we assessed publicly available shotgun metagenomics processing packages/pipelines including bioBakery, Just a Microbiology System (JAMS), Whole metaGenome Sequence Assembly V2 (WGSA2), and Woltka using 19 publicly available mock community samples and a set of five constructed pathogenic gut microbiome samples. Also included is a workflow for labelling bacterial scientific names with NCBI taxonomy identifiers for better resolution in assessing results. The Aitchison distance, a sensitivity metric, and total False Positive Relative Abundance were used for accuracy assessments for all pipelines and mock samples. Overall, bioBakery4 performed the best with most of the accuracy metrics, while JAMS and WGSA2, had the highest sensitivities. Furthermore, bioBakery is commonly used and only requires a basic knowledge of command line usage. This work provides an unbiased assessment of shotgun metagenomics packages and presents results assessing the performance of the packages using mock community sequence data.

## Introduction

The field of bioinformatics analysis for microbiome research is progressing at a rapid pace and computational tools are being continuously developed and updated to profile the composition and taxonomy of microbial communities^1,2^. Unlike 16S ribosomal RNA (16S rRNA) gene sequencing, which is comprised of short read amplicons, shotgun metagenomics sequencing (SMS) is defined as a high-throughput method used to study the genetic composition of the microorganisms present in a human microbiome sample^3^. With 16S rRNA gene sequencing taxonomy classification, workflows are generated from denoised or clustered amplicon sequences that target only the 16S hypervariable regions of the bacteria genome against standard reference databases such as Silva, Greengenes or the Ribosomal Database Project^4–6^. Alternatively, shotgun metagenomics profiling can be performed using different profiling options, sometimes including assembly of the short reads into longer fragments called contigs. To generate the databases of known isolates, some bacterial species can be cultivated to obtain isolate genomes, but many species are not amenable to being cultured, especially in environmental samples^7^. Despite this issue, many new approaches have allowed researchers to recover genomes from metagenomic samples in what are known as metagenome assembled genomes (MAGs), bypassing the need to culture in the lab^8^. The concept surrounding the assembled genomes is that the MAGs are binned into similar bacterial species or strains based on sequence similarity characteristics or coverage^9^. There are large scale databases created specifically for the purpose of utilizing MAG workflows which are being curated at an increasing rate^10–12^. These databases are utilized successfully in conjunction with reference-based approaches for taxonomic classification. One commonly used shotgun metagenomics tool called MetaPhlAn4 utilizes $\widetilde{{\rm{1}}}$.01 million prokaryotic MAGs and isolate genomes for taxonomic assignment^13^.

Shotgun metagenomics profiling is an attractive method for researchers since one of its strengths as compared to 16S rRNA sequencing is in the specificity of characterizing species-level bacteria within the sample^14^. Additionally, shotgun metagenomics sequencing also allows for the assessment of other parts of the bacterial genome besides the rRNA gene of the microbe. Species-level classification has become more important in the microbiome research field due to its relevance in human health and in clinical applications^15^. However, assessing the species-level taxonomic classification capability can present a challenging problem between taxonomic profilers. Thus, several shotgun metagenomics pipelines have been developed, including the Whole MetaGenome Sequence Assembly pipeline (WGSA2)^16^, Just A Microbiology System (JAMS)^17^, Sunbeam^18^, and MEDUSA^19^. Both WGSA2 and JAMS use Kraken2^20^, a k-mer based classifier. In 2022, a more recently introduced classifier, Woltka, uses an operational genomic unit (OGU) approach and is based on phylogeny, which utilizes the evolutionary history of the species lineage^21^. Furthermore, another approach to profiling shotgun metagenomics sequencing is a marker gene approach developed by the Huttenhower Lab. The marker gene approach, which can be utilized within the bioBakery suite of bioinformatics tools, is referred to as Metagenomic Phylogenetic Analysis (MetaPhlAn). As of 2023, there are three versions of MetaPhlAn; both versions two (MetaPhlAn2) and three (MetaPhlAn3) are marker gene-based, while version four (MetaPhlAn4) is both marker gene and MAG-based^13,22,23^. The most important distinction is that both MetaPhlAn3 and MetaPhlAn4 utilize the marker-gene approach, but MetaPhlAn4 critically incorporates the metagenome assembled genomes into its classification scheme. This is to make up for the weakness of MetaPhlAn3, where organisms that were not included in the reference genome were unable to be detected. Instead MetaPhlAn4 utilizes the species-genome bins (SGBs) as the base unit of classification. Some known bins, such as those in the reference genome, remain as they were and are instead called known species-level genome bins (kSGBs) and the newly-assembled ones that are not present in the reference databases (but are approximately species level) are called unknown species-level genome bins (uSGBs). Altogether, MetaPhlAn4 can provide more granular, less “strict” classification than MP3. The pipelines also differ in their assembly protocols since genome assembly is always performed in JAMS whereas genome assembly is optional in WGSA2. Assembly is not performed at all in the Woltka classifier.

While new tools are consistently being developed for taxonomic classification and profiling in microbiome studies, the end user is left with the question of which package is the most optimal bioinformatics processing package for their shotgun metagenomics analyses and preferences. Because most tools and pipelines do not include objective classification accuracy data in the source documents as a default, this can further complicate the selection of SMS processing tools given potential performance variability based on a researcher’s specific question or microbiome community of interest. A useful tool for benchmarking assessment is mock bacterial communities, which are curated microbial communities generated with known, ground truth compositions of bacterial species or strains. These known communities can be generated either computationally or cultured in the lab to test the accuracy of bioinformatics pipelines^24,25^. Microbiome sample preparation can include biases at any given stage of a microbiome study from sampling method, DNA extraction, and choice of sequencing instrument, among others can be introduced at different stages in the microbiome prepping protocol^26–29^. Mock bacterial communities can be used to assist with the biases in mind in order to benchmark sequencing and profiling protocols. There has been much effort on benchmarking pipelines for 16S rRNA sequencing^30^, leaving a relative dearth of shotgun metagenomics mock community benchmarking. Fortunately, more shotgun-based mock bacterial community sequences have become available with the decreasing cost and increased utilization of shotgun metagenomics sequencing methods. These shotgun sequences of standard mock communities range from sequenced data extracted from whole bacterial cells to simulated sequences of microbial communities^25,31–34^.

Benchmarking studies that assess SMS mock bacterial communities are valuable as they provide comparisons of accuracy and precision metrics across taxonomy classifier pipelines, but deciding which pipeline to use still poses a challenge^33,35–37^. First, these studies are usually tested in a proof of concept of the standards or mock communities themselves or used to benchmark a bioinformatics tool created by the research team publishing the work. Although these studies usually provide detailed documentation of their benchmarking and testing strategies, there may be some unintentional bias in the metrics and results reported given the authors and research team are invested in the success of the outcome measure that is being tested. The Critical Assessment of Metagenome Interpretation (CAMI) challenge consortium was a foundational effort across the global software developer and bioinformatics community to evaluate SMS processing pipelines and parameters^38^. Although the CAMI challenge results provided comprehensive data to assist in the selection of SMS assemblers and taxonomic profilers, many of the tools evaluated in the CAMI challenge have since been updated (i.e., MetaPhlAn2). As the goal of the effort was to standardize the input sequences used across the benchmarking teams, the complex metagenome benchmarking datasets used in the benchmarking assessments was artificially generated to include both representative characteristics of metagenomes generated from a microbiome study, along with genomes of organisms that would be more challenging to classify using standard methods and public databases^38^. Subsequent benchmarking studies have evaluated the performance of pipelines and classifiers using analyses focused on classification accuracy^35,39,40^, read length^41^, sequencing depth^42^, and methodology^43,44^. Although all of these benchmarking studies have contributed valuable data to guide the selection of tools for SMS analysis, there are newly developed annotation pipelines and profilers that have been published in microbiome research studies, but have not yet been benchmarked in the currently published literature. This manuscript intends to perform an objective benchmarking analysis of these recent pipelines and profilers using several different mock bacterial community and metagenome benchmarking datasets to contribute to the literature and to fill this gap. Another challenge often overlooked in microbiome studies, with the highly variable taxonomic naming schemes for (but not only) bacterial taxa across reference databases. This poses an issue in comparing results across databases or merging at a higher level of taxonomy without a significant amount of data wrangling and naming reclassification (see Maki *et al*., for methods used to merge at the genus level for 16S rRNA taxonomy^45^). A resolution to this problem was sought by the National Center for Biotechnology Information (NCBI), who proposed NCBI taxonomy identifiers (TAXIDs). Since names do not remain static, two previously separate organisms can be merged into one, and organisms can be reclassified into other genera. TAXIDs provide a single, unified way to unambiguously identify organisms across several pipelines and naming schemes^46^. As reference databases for both 16S rRNA amplicons and shotgun sequences are frequently updated with new taxonomy names, a consistent problem in benchmarking studies exists with linking retired taxonomy names across multiple workflows. This issue can be resolved by generating taxonomy identifiers from scientific names in a programmatic fashion, which was integrated into the overall assessment workflow of this benchmarking study.

Finally, microbiome sequencing data is compositional by nature, and many of the distance metrics used (i.e., UniFrac or Bray-Curtis distance measures) do not account for the constraints and assumptions of compositional data matrices^47^. Consequently, there is a lack of published benchmarking studies using compositional distance metrics, like Aitchison Distance (AD), despite the field of microbiome science encouraging compositionally-aware analysis metrics in published research^48^. As reporting standards and preferred analysis metrics continue to be defined, non-compositional distance metrics may be reported less frequently which will make cross-benchmarking study comparison challenging. In this study, we chose to focus on four pipelines. First, the bioBakery suite was chosen because it is well known and often used in microbiome analysis workflows. WGSA2 and JAMS were both developed at the National Institutes of Health, and were considered for this work because there has been no validation performed on these two pipelines in the literature and also as side-by-side comparisons due to their similar profiling methods, but widely varying downstream capabilities. Finally, Woltka was included because it is relatively newer (published April 2022) and is a phylogeny-aware classifier and is quite different than the other three pipelines considered. The aim of this work was to perform an assessment of select shotgun metagenomics packages and pipelines, especially with adherence to AD as the preferred metric to assess compositional closeness to the expected compositions.

## Results

### Overview of the mock community samples

This study includes 24 mock community samples that were submitted to four shotgun metagenomics processing pipelines, one of which included two versions of the same pipeline. The samples were sourced from previous publications and are referred to as BMock12^33^, CAMISIM^38^, Amos HiLo, Amos Mixed^32^, and Tourlousse^31^. The other mock samples not sourced from previous publications were constructed and sequenced specifically for this study by the National Institute of Standards and Technology (NIST). The majority of the samples (23 of 24) consisted of gut microbiome organisms, and one was constructed based on environmental and aquatic organisms (BMock12). Additionally, two of the gut microbiome samples were generated from simulated data (CAMISIM). The BMock12, CAMISIM, and NIST samples did not contain technical replicates and are grouped in the category called the “One-to-One” communities, meaning one sample matches to one separate expected composition. The Amos and Tourlousse communities included technical replicates (5 for each Amos community and 6 for Tourlousse), and therefore are referred to as the “Replicate” communities. Overall, the expected relative abundances for all mock communities are shown in Supplementary Table S1a–f.

### Read statistics of all mock community samples

**Fig. 1:**
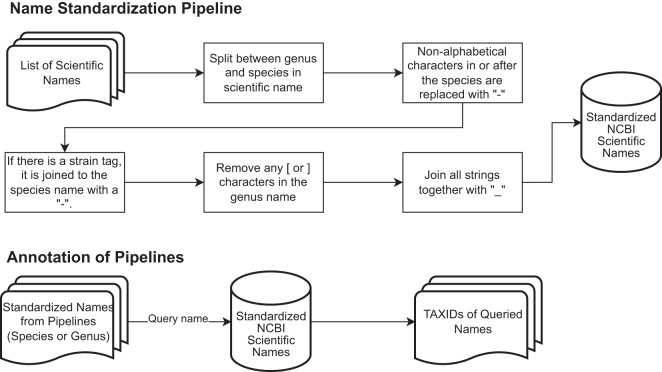
A flowchart describing the process of name standardization. The top portion summarizes how the standardized NCBI Taxonomy database was compiled. The bottom portion illustrates how scientific names were queried in the standardized NCBI database to obtain TAXIDs.

The Illumina Sequencing platform was used for all shotgun metagenomics sequencing of all mock bacterial communities. The total number of sequencing reads per sample (1.9 to 100.5 million), average read length (84bp to 200bp) and type of Illumina sequencing platform is summarized in Table 1. The pipelines assessed include: two versions of bioBakery workflows, JAMS^17^, WGSA2^16^, and Woltka^21^. Since bioBakery workflows was run with either MetaPhlAn3 (bioBakery3) or MetaPhlAn4 (bioBakery4)^13,23^, these were assessed as separate pipelines in this manuscript and a flow chart depicting the taxonomic schema for resolving names for this project is shown in Fig. 1.

**Table 1:** Summary of read statistics for the communities of interest.

	Community Mean ± SD	n	Number Sequences (Millions)	Average Read Length	Max–Min Read Length	Sequencing Platform
***One-to-One***	BMock12	1	100.5	151.00 ± 0.00	205–100	HiSeq 2500
	S1 CAMISIM	1	16.67	148.45 ± 0.78	150–31	Simulated
	S2 CAMISIM	1	16.67	148.45 ± 0.78	150–31	Simulated
	EG NIST	1	3.35	84.10 ± 1.13	151–15	MiSeq
	Mix-A NIST	1	3.47	105.35 ± 0.78	151–19	MiSeq
	Mix-B NIST	1	3.58	105.05 ± 0.78	151–18.5	MiSeq
	Mix-C NIST	1	2.97	109.55 ± 0.78	151–16.5	MiSeq
	Mix-D NIST	1	3.28	113.85 ± 0.64	151–20	MiSeq
***Replicates***	Amos HiLo	5	1.90 ± 0.17	200.54 ± 1.93	151–151	NextSeq 500
	Amos Mixed	5	2.01 ± 0.18	200.71 ± 1.55	151–15	NextSeq 500
	Tourlousse	6	5.90 ± 0.38	145.12 ± 1.31	205–100	NextSeq 500

The One-to-One communities have standard deviations because the forward and reverse reads were averaged together.

### Relative abundances of all mock community samples

**Fig. 2:**
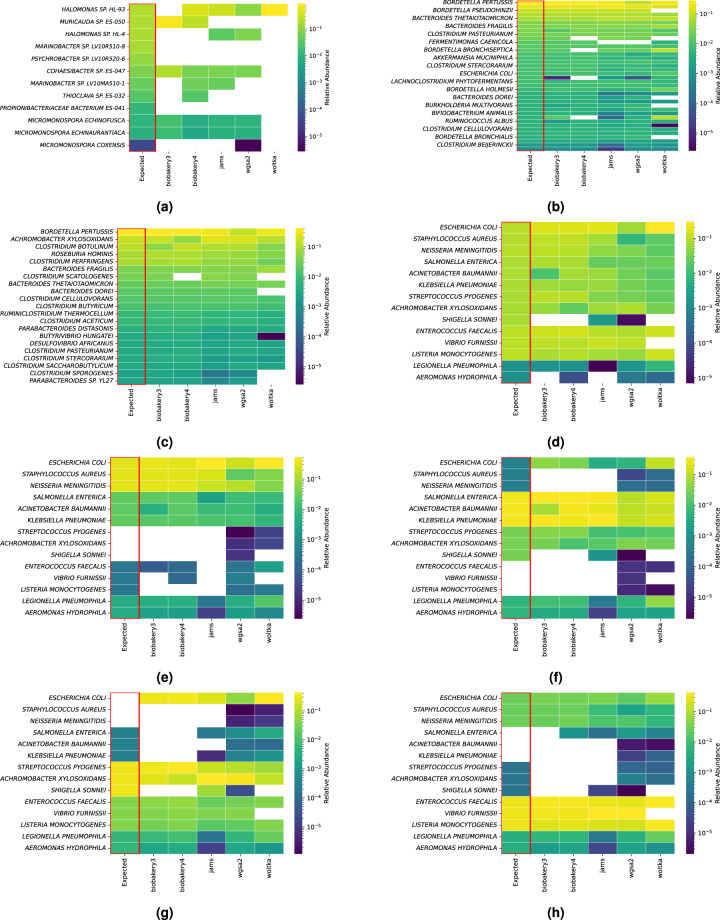
Heatmaps of expected taxa (y-axis) and observed output from each pipeline (x-axis) for each One-to-One community. Each heatmap shows the logarithm of relative abundances of both expected (first column) and observed for the following samples (**a**) BMock12, (**b**) S1 CAMISIM, (**c**) S2 CAMISIM, (**d**) NIST-EG, (**e**) NIST-A, (**f**) NIST-B, (**g**) NIST-C, (**h**) NIST-D. Missing cells indicate a relative abundance of 0. The species listed in Fig. 2c shows only half of the species names due to spacing constraints. These were created from a left join on pipeline output before any filtering, where the expected species are placed on the left axis and compared to the output of the pipelines. No unexpected species are displayed.

**Fig. 3:**
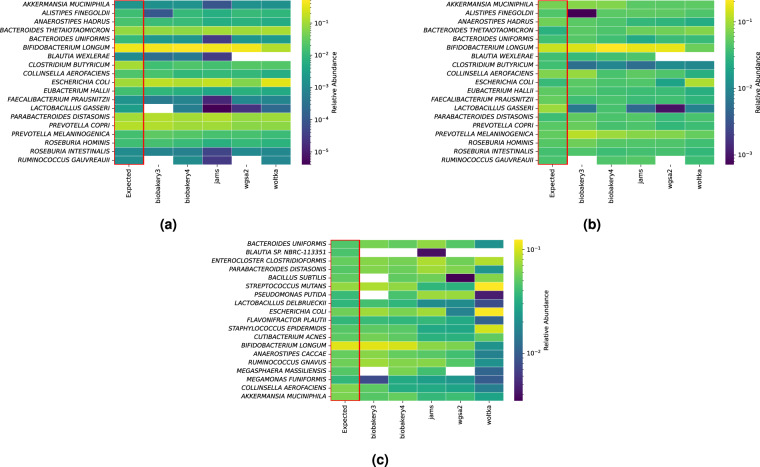
Heatmaps of expected taxa (y-axis) and observed output from each pipeline (x-axis) for each Replicate community. Each heatmap shows the logarithm of relative abundances for both expected and observed for the following replicates (**a**) Amos HiLo, (**b**) Amos Mixed and (**c**) Tourlousse. Missing cells indicate a relative abundance of 0. One sample from each replicate group is shown for conciseness since all the observed relative abundances for all replicates were nearly identical. These were created from a left join on pipeline output before any filtering, where the expected species are placed on the left axis and compared to the output of the pipelines. No unexpected species are displayed.

Results will refer to the mock communities as either One-to-One or Replicates communities. These descriptors refer to whether or not the community used did not include any technical replicates (One-to-One) or if the community assessed did include technical replicates (Replicates). To be more specific, the BMock12, CAMISIM, and NIST samples dwill be referred to the “One-to-One” communities because they were standalone samples assessed. The Amos and Tourlousse communities included technical replicates (5 for each Amos community and 6 for Tourlousse) and will be referred to as the “Replicate” communities. The expected relative abundance values (RA) are visualized against the observed RA values in heatmaps for both the One-to-One and the Replicate communities in Figs. 2 and 3, respectively. No filtering was performed on the data shown in the heatmaps. Missing cells in the heatmaps indicate that the pipeline did not identify any reads of the expected bacterial species for that mock community. While useful as a qualitative assessment of performance, quantitative analysis is performed in the “Accuracy Assessment” section.

#### One-to-One mock community heatmaps

The One-to-One community heatmaps are shown in Fig. 2. The BMock12 community (Fig. 2a) shows the heatmap of the expected (logarithm transformed) RA values compared to the observed RA values for each pipeline. For the BMock12 community, of the 12 expected species, there were three species that were not detected by any pipeline including *Marinobacter sp. LV10R510-8*, *Psychrobacter sp. LV10R520-6* and *Propionibacteriaceae bacterium ES-041*. Alternatively, there were four other species that were detected by four of the five pipelines including *Halomonas sp. HL-93*, *Cohaesibacter sp. ES-047*, *Micromonospora echinofusca* and *Micromonospora echinoaurantiaca*. For the remaining 5 species, *Muricauda sp. ES.050* was detected by bioBakery3 and bioBakery4, *Halomonas sp. HL-4* by JAMS and WGSA2, *Marinobacter sp. LV10MA510-1* by bioBakery4 and JAMS, *Thioclava sp. ES.032* by bioBakery4, and *Micromonospora coxensis* by WGSA2.

The CAMISIM mock community, composed of samples 1 and 2, is shown in Fig. 2b,c, respectively. All 38 species were identified in bioBakery3 (2). JAMS and WGSA2 both only missed *Fermentimonas caenicola*, while bioBakery4 missed identifying four species: *Clostridium scatologenes, Bordetella bronchiseptica, Eubacterium limosum* and *Bordetella parapertussi*. Also, Woltka was unable to correctly identify 5 of the expected species in sample 1: *Bordetella pseudohinzii, Clostridium scatologenes, Bacteroides dorei, Burkholderia multivorans* and *Bordetella bronchialis*. Next, sample 2 of CAMISIM is shown in 2. All 21 species from this sample were correctly identified using bioBakery3, JAMS and WGSA2. However, bioBakery4 was unable to identify *Clostridium scatologenes*. Woltka was unable to find four of the 21 species: *Clostridium scatologenes, Bacteroides dorei, Clostridium sporogenes* and *Parabacteroides sp. YL27*.

The five constructed NIST samples (NIST-EG, NIST-A, NIST-B, NIST-C and NIST-D) are shown in Fig. 2d–h. Of the NIST samples, the even sample (EG), which had approximately equal concentrations for each organism (Fig. 2d) had only six missed species. When assessing the performance of the staggered communities, which have unequal concentrations of each organism (Fig. 2e–h), only WGSA2 was able to discern all expected species in all of the NIST samples. Woltka missed 2 species *Shigella sonnei* and *Vibrio furnissii* for all 4 staggered samples. Finally, bioBakery3, bioBakery4 and JAMS each missed at least 3 species for any of the 4 staggered samples.

#### Replicate community heatmaps

Figure 3 shows a heatmap of one sample from each of the Replicate communities (Amos HiLo, Amos Mixed, Tourlousse). The heatmaps shown here represent one replicate from each group. Representative Amos HiLo and Amos Mixed heatmaps (Fig. 3a,b) demonstrate all 19 species were identified by two of the five pipelines (JAMS and bioBakery4). *Blautia wexlerae* was not identified by WGSA2 or Woltka for both the HiLo and the Mixed community. Furthermore, *Ruminococcus gauvreauii* was not found by bioBakery3 and WGSA2 for both the HiLo and the Mixed community. One species, *Lactobacillus gasseri* was not found for the Amos HiLo sample by bioBakery3 but interestingly this species was found in the Amos Mixed community by bioBakery3.

Finally, Fig. 3c shows one replicate from the Tourlousse samples. All 19 species were identified by JAMS. The species *Blautia sp. NBRC-113351* was not identified by any pipeline except JAMS. Shown in the heatmaps, bioBakery3 failed to identify 4 species out of 19: *Blautia sp. NBRC-113351, Bacillus subtilis, Pseudomonas putida, Megasphaera massiliensis*. WGSA2 failed to identify *Megasphaera massiliensis* in addition to *Blautia sp. NBRC-113351*.

### Accuracy assessment of each pipeline

**Fig. 4:**
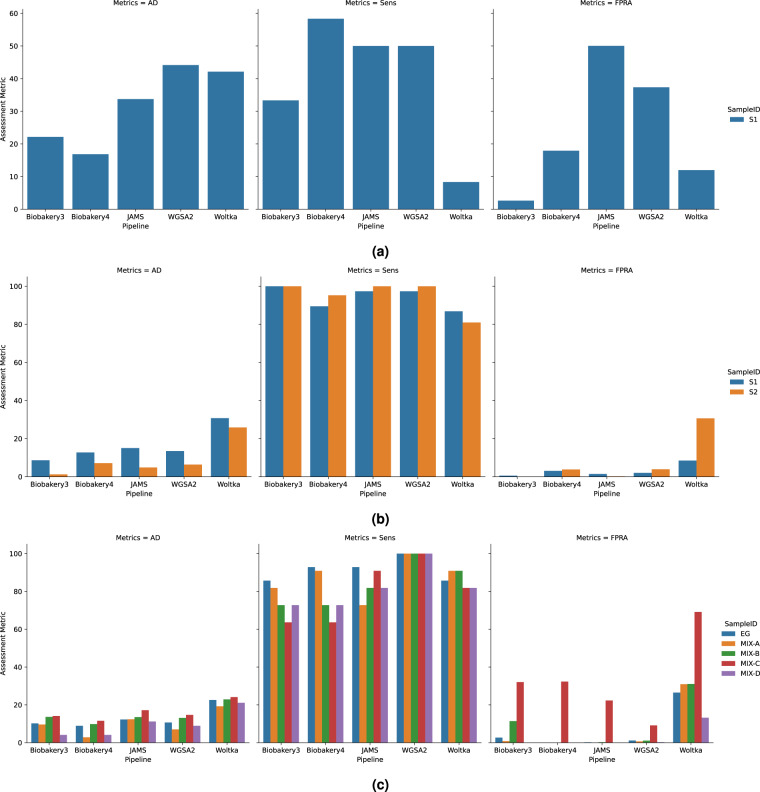
Assessment metrics bar plots for the One-to-One communities of (**a**) Bmock12, (**b**) CamiSim sanples S1 (blue) and S2 (orange) and (**c**) NIST samples EG (blue), Mix-A (orange), Mix-B (green), Mix-C (red) and Mix-D (purple). Each panel is subdivided by the 3 assessment metrics as follows: Aitchison Distance (AD), Sensitivity metric (Sens) and False Positive Relative Abundance (FPRA). Each pipeline is shown on the x-axis. Averages of each assessment metric across all samples were assessed using a Kruskal-Wallis (KW) test and are reported in Tables 2–4. Results of KW test overall are as follows: AD: *p* < 0.001; Sens: *p* = 0.133; FPRA: *p* = 0.001.

**Fig. 5:**
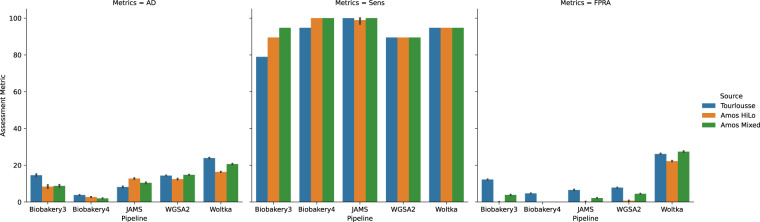
Assessment metrics bar plots for the Replicate communities. Bars indicate the averaged metric for each replicate community for Amos Mixed (blue), Amos HiLo (orange) and Tourlousse (green) using Aitchison Distance (AD), Sensitivity (Sens) and False Positive Relative Abundance (FPRA). The panel is subdivided by the assessment metric result. Standard error bars are shown for each replicate within each set of samples. Average Assessment metric is plotted. Averages of each assessment metric across all samples were assessed using a Kruskal-Wallis (KW) test and are reported in Tables 2–4. Results of KW test overall are as follows: AD: *p* < 0.001; Sens: *p* = 0.133; FPRA: *p* = 0.001.

The accuracy metrics reported in this report are used to assess how well a pipeline performed at approximating the mock community sample of interest. The metrics used are AD, Sensitivity, and False Positive Relative Abundance (FPRA). The accuracy metrics for the one-to-one samples are shown in Fig. 4 and metrics for the Replicates group are shown in Fig. 5. All metrics are tabulated in Tables 2, 3, and 4.

**Table 2:** Table of Aitchison Distance metrics.

Aitchison Distance													
	Sample Type	n	bioBakery3		bioBakery4		JAMS		WGSA2		Woltka		Average
			Mean	Stdev	Mean	Stdev	Mean	Stdev	Mean	Stdev	Mean	Stdev	
***One-To-One***	Bmock12	1	22.15		16.83		33.72		44.16		42.14		31.80
	CAMISIM-S1	1	8.62		12.76		15.04		13.48		30.76		16.13
	CAMISIM-S2	1	1.23		7.11		4.85		6.38		25.88		9.09
	NIST-EG	1	10.18		8.94		12.25		10.66		22.60		12.93
	NIST-MIX-A	1	9.59		2.84		12.35		7.00		19.24		10.20
	NIST-MIX-B	1	13.65		9.81		13.55		13.08		22.88		14.59
	NIST-MIX-C	1	14.11		11.57		17.16		14.70		24.10		16.33
	NIST-MIX-D	1	4.13		4.11		11.22		8.93		21.12		9.90
***Replicates***	Amos HiLo	5	8.40	1.07	2.71	0.04	12.80	0.34	12.44	0.29	16.38	0.11	10.55
	Amos Mixed	5	8.77	0.77	2.00	0.06	10.42	0.36	14.82	0.05	20.63	0.22	11.33
	Tourlousse	6	14.57	0.73	3.81	0.02	8.16	0.40	14.38	0.10	23.90	0.26	12.96
**Average**	*p* < 0.001		10.49	5.60	7.50	4.89	13.77	7.38	14.55	10.27	24.51	6.92	

A perfect score is 0 (i.e., perfect agreement between expected and observed). A higher average AD value means a community is more difficult to classify. AD’s were significantly different across all pipelines and samples (*p* < 0.001, Kruskal-Wallis).

**Table 3:** Table of Sensitivity metrics.

Sensitivity													
	Sample Type	n	bioBakery3		bioBakery4		JAMS		WGSA2		Woltka		Average
			Mean	Stdev	Mean	Stdev	Mean	Stdev	Mean	Stdev	Mean	Stdev	
***One-to-One***	Bmock12	1	33.33		58.33		50.00		50.00		8.33		40.00
	CAMISIM-S1	1	100.00		89.47		97.37		97.37		86.84		94.21
	CAMISIM-S2	1	100.00		95.24		100.00		100.00		80.95		95.24
	NIST-EG	1	85.71		92.86		92.86		100.00		85.71		91.43
	NIST-MIX-A	1	81.82		90.91		72.73		100.00		90.91		87.27
	NIST-MIX-B	1	72.73		72.73		81.82		100.00		90.91		83.64
	NIST-MIX-C	1	63.64		63.64		90.91		100.00		81.82		80.00
	NIST-MIX-D	1	72.73		72.73		81.82		100.00		81.82		81.82
***Replicates***	Amos HiLo	5	89.47	0.00	100.00	0.00	98.95	2.35	89.47	0.00	94.74	0.00	94.53
	Amos Mixed	5	94.74	0.00	100.00	0.00	100.00	0.00	89.47	0.00	94.74	0.00	95.79
	Tourlousse	6	78.95	0.00	94.74	0.00	100.00	0.00	89.47	0.00	94.74	0.00	91.58
**Average**	*p* = 0.133		79.37	19.21	84.60	14.94	87.86	15.57	92.34	14.81	81.05	24.70	

A perfect score is 100 (i.e., all expected species were identified). A lower average sensitivity value means a community is more difficult i.e., expected species were not identified). Sensitivity metrics were not significantly different across all pipelines and samples (*p* = 0.133, Kruskal-Wallis).

**Table 4:** Table of False Positive Relative Abundance metrics.

FPRA													
	Sample Type	n	Biobakery3		Biobakery4		JAMS		WGSA2		Woltka		Average
			Mean	Stdev	Mean	Stdev	Mean	Stdev	Mean	Stdev	Mean	Stdev	
***One-to-One***	BMock12	1	2.63		17.93		50.04		37.34		11.96		23.98
	CAMISIM S1	1	0.53		3.06		1.44		2.04		8.51		3.11
	CAMISIM S2	1	0.06		3.83		0.21		3.91		30.67		7.74
	NIST EG	1	2.66		0.00		0.21		1.16		26.48		6.10
	NIST MIX-A	1	0.85		0.00		0.05		0.68		30.92		6.50
	NIST MIX-B	1	11.42		0.01		0.27		1.09		31.06		8.77
	NIST MIX-C	1	32.06		32.31		22.33		9.16		69.17		33.01
	NIST MIX-D	1	0.06		0.10		0.02		0.25		13.19		2.73
***Replicates***	Amos HiLo	5	0.01	0.02	0.00	0.00	0.19	0.07	0.73	0.08	22.24	0.21	4.63
	Amos Mixed	5	3.86	0.22	0.00	0.00	2.13	0.12	4.47	0.10	27.39	0.31	7.57
	Tourlousse	6	12.23	0.14	4.72	0.04	6.56	0.32	7.82	0.05	26.14	0.25	11.49
**Average**	*p* = 0.001		6.03	9.68	5.63	10.31	7.59	15.56	6.24	10.74	27.07	16.14	

A perfect score is 0 (i.e., no false positive values were identified). A higher average FPRA value means a community is more difficult (i.e., high abundance of falase positive species identified). FPRA’s were significantly different across all pipelines and samples (*p* = 0.001, Kruskal-Wallis). The p-value was calculated using the Kruskal-Wallis test across the five groups.

**Fig. 6:**
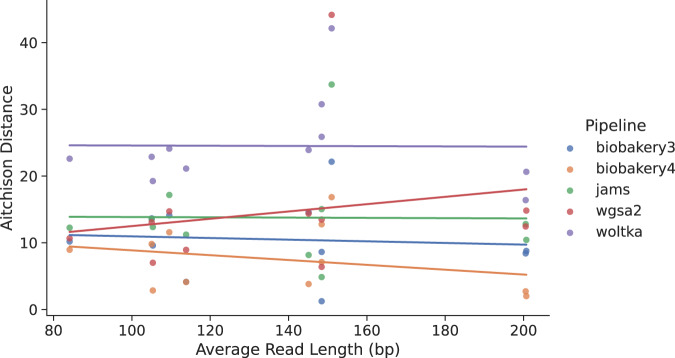
Bivariate Y by X plot of Aitchison distance (y-axis) compared to the average read length (x-axis) for each sample in each pipeline. Correlation between AD and average read length was assessed using Pearson Correlation. Pearson correlation coefficients: bioBakery3 (blue, n= 9, *r* = −0.08, *ρ* = 0.805), bioBakery4 (orange, n = 10, *r* = −0.28, *ρ* = 0.396), JAMS (green, n = 11, *r* = −0.01, *ρ* = 0.975), WGSA2 (red, n = 11, *r* = −0.20, *ρ* = 0.546), Woltka (purple, n = 11, *r* = −0.01, *ρ* = 0.979).

In order to assess how the accuracy metrics differed between the pipelines, the overall averages were compared using a Kruskal-Wallis test. There was a significant difference of the AD across all pipelines (Table 2, H = 25.92, *p* < 0.001) suggesting that all pipelines performed differently. When the AD of each pipeline was considered individually, bioBakery4 has the lowest average AD over all communities (7.50 ± 4.89). Next, bioBakery3 performed second best with an average AD of 10.49 ± 5.60. JAMS and WGSA2 performed closely with an average AD of 13.77 ± 7.38 and 14.55 ± 10.27, respectively. Woltka had the highest average AD of 24.51 ± 6.92. The post-hoc pairwise Wilcoxon tests (all permutations) with p-values are tabulated in Supplementary Table S2a. Since one might hypothesize that a longer read length might contribute to a better AD (i.e., longer reads can increase accuracy and therefore contribute to a closer representation of what is in the sample), the AD and read lengths were assessed by correlation. There was a slight negative correlation when comparing the AD values to the average read length for all pipelines except for WGSA2, however, none were significant (Fig. 6). The Pearson correlation coefficients for each pipeline are as follows: bioBakery3 (*r* = −0.08, *p* = 0.805), bioBakery4 (*r* = −0.28, *p* = 0.396), JAMS (*r* = −0.01, *p* = 0.975), WGSA2 (*r* = 0.20, *p* = 0.546), Woltka (*r* = −0.01, *p* = 0.979) indicating that read length did not show any significant contribution to accuracy for this data.

The sensitivity metric captures the total number of true positive species found over the total expected species. Over all pipelines, sensitivities ranged from 79.65–91.93% (Table 3). When the average sensitivity was considered across all pipelines, there was no significant differences observed (*H* = 7.06, *p* = 0.13). On average, however, WGSA2 had the highest average sensitivity value (92.34 ± 14.81%), followed by JAMS (87.86 ± 15.57%), bioBakery4 (84.60 ± 14.94%), Woltka (81.05 ± 24.70%), and finally bioBakery3 (79.37 ± 19.21%). The pairwise Wilcoxon tests are tabulated in Supplementary Table S2b.

Finally, when assessing the FPRA, which quantifies the total number of false positive relative abundances, a significant difference was observed across all pipelines using the Kruskal-Wallis test (*H* = 17.89, *p* = 0.001). All FPRA metrics can be observed in Table 4. Across the communities, a similar trend to the AD results was observed. As before, bioBakery4 performed the best with the lowest average FPRA of 5.63 ± 10.31%, then bioBakery3 at 6.03 ± 9.68%, WGSA2 at 6.24 ± 10.74%, JAMS at 7.59 ± 15.56%, and finally Woltka at 27.07 ± 16.14%.

Unclassified reads can also be a useful metric to assess the classification strength of a pipeline. The number of unclassified reads from the Kraken2 methods in this study were assessed; however, those for the bioBakery tools or Woltka were not since they are not provided by default. The MetaPhlAn classifiers will only return high confidence results as it searches for matching alignments, rather than annotating every sequence. Furthermore, Woltka also does not provide the number unclassified by default. Finally the average of unclassified for JAMS was 4.74 ± 4.17 and WGSA2 was 5.12 ± 8.85. These results are tabulated in Supplementary Table S8.

### Binary classification performance of NIST community using a confusion matrix

Confusion matrices can be used to represent a prediction summary in matrix form and were used in this study to assist in determining the classification performance of the pipelines, i.e., specificity and sensitivity^49^. The confusion matrix quantification was applied to the set of NIST samples since there were separate samples with added and removed organisms over five mock community constructs. This allowed for several compositions with varying true positive and true negative bacterial species across the five samples. More detailed methods of this application can be found in the Methods section. The overall resultsof the analysis and interpretation of the confusion matrice application yielded two separate trends depending on the threshold chosen. At 0% filtering (i.e., any amount of RA was considered a "hit") Woltka showed the highest harmonic mean F1 score at 0.91, closely followed by WGSA2 at 0.90. JAMS and bioBakery4 performed identically with the same harmonic mean F1 score of 0.89. Lastly, bioBakery3 had a harmonic mean F1 of 0.88. When a slightly more rigorous filter was applied of 0.01%, (i.e., any RA values of the observed taxa that were below .01% were removed before calculations), WGSA2 performed best with an F1 score of 0.97. Woltka was very close with a score of 0.96. Then in consecutive order, bioBakery4 had an F1 score of 0.88, bioBakery4 0.87 was fourth, and finally JAMS at 0.86 was last. The results are tabulated in Supplementary Tables S4a, S5e and can be visualized in Supplementary Figures S1, S2.

The confusion matrix was constructed to assess the performance of accuracy by each organism for the NIST samples (Supplementary Tables S4a, S5e). JAMS was able to correctly assign *Shigella sonnei* above a 0.01% filtering threshold, since there were zero true positives in bioBakery3, bioBakery4, WGSA2, and Woltka. At 0% filtering, JAMS and WGSA2 could identify *Shigella sonnei*. Furthermore, *Vibrio furnissii* was missed by Woltka at both 0% and 0.01%, and *Aeromonas hydrophila* by JAMS only at 0.01%. More specifically, at the 0.01% filtering threshold, bioBakery3 showed about the same F1 score (≈ 0.85) for all species. It was most sensitive at identifying *Legionella sp*. with a 100% true positive rate. However, note that this was one of the controls, which means it was included in all samples. However, bioBakery3 did fail to detect *Shigella sp*. and had a 0% sensitivity. Finally, *E. coli* had a 0% specificity score, meaning the pipeline reported it was always present in every sample.

Overall, bioBakery4 performed slightly better by harmonic mean than bioBakery3 (0.88 vs 0.87, respectively). Improvements were seen in detection of *Enterococcus sp*. and *Vibrio sp*. to 100% sensitivity. However, the F1 score of *Salmonella sp*. decreased from 0.86 to 0.75 in bioBakery4. Poor specificity (0%) was seen in *E. coli* and *Salmonella sp*., which still evaded detection from bioBakery4. JAMS had only one outlier, with the rest performing very close to the harmonic mean F1 score. JAMS failed to detect *Aeromonas sp*. in any of the samples. JAMS also had low specificity for *E. coli* and *Salmonella sp*. with 0% each. WGSA2 performed the best when comparing F1 harmonic means (0.97) with excellent sensitivity across most species. However, WGSA2 suffered from low specificity for *E. coli* and *Salmonella sp*, which WGSA2 failed to detect. Finally, Woltka performed very well when considering harmonic mean F1 scores (0.96) with excellent sensitivity across almost all species. However, Woltka failed to detect *Shigella sp*. and *Vibrio sp*., and it also had low specificity for *E. coli*, *Klebsiella sp*., and *Salmonella sp*.

### Ease of use of each pipeline

When considering 'ease of use' of the pipelines surveyed, bioBakery is the most commonly cited in current microbiome research, has extensive documentation (https://github.com/biobakery/biobakery/wiki/biobakery_workflows), a large community forum, and is the most compatible across many hardware configurations. The MetaPhlAn3 paper has, at the time of writing this manuscript, 310 citations and the MetaPhlAn4 paper has one citation. It has several installation methods through both Python Anaconda, pip, and Docker. Furthermore, bioBakery workflows only requires a basic knowledge of command line interface (CLI) to use effectively.

WGSA2, through the online platform NEPHELE, requires manual input and generation of each metadata file with no backend application programming interface  for automation, and may require long upload times if certain file transferring tools, i.e., Globus (https://www.globus.org/), are not available. However, WGSA2 is a great choice for a novice user since it is available through a web browser and provides cloud-based computational resources to utilize the service. Researchers with minimal computing resources or limited command-line interface knowledge may prefer WGSA2 for their microbiome analysis needs. NEPHELE is also well-supported and includes a very nice tutorial with extensive documentation (https://nephele.niaid.nih.gov/user_guide_tutorials/). NEPHELE is also well-supported with staff available to answer questions for users.

JAMS is currently only executable on the Biowulf super-computer cluster at the National Institutes of Health (https://hpc.nih.gov/) but at the time of writing, JAMS is being adapted to be run outside of the Biowulf HPC computing environment. Effective usage of JAMS requires CLI knowledge and customizing the plotting functions require a working understanding of the R programming language.

Finally, Woltka is available through both Python pip and Anaconda without operating system restrictions. However, the Woltka classifier is not a complete workflow and does not provide further analyses or visualizations; thus, this would require the user to utilize another platform or the integrated Quantitative Insights Into Microbial Ecology (QIIME2) plugin to perform the downstream analysis separately with the output in tab-separated-values or BIOM formats. Furthermore, the user must perform the alignment against a reference database prior to running the classification, which adds other decisions that must be made for the user to build the profiling workflow.  This tool would require the user to have a very extensive bioinformatics knowledge in order to perform the needed microbiome analysis.

## Discussion

The aim of this work was to assess the species-level compositional structure of known mock community samples using different shotgun metagenomics processing pipelines which rely on three different profiling approaches: k-mer based approach (Kraken2), unique clade-specific marker genes, or an operational genomic unit (OGU) approach. As part of this workflow, a taxonomic identifier linking methodology was built such that no ambiguity of species names arose between pipelines and species names from each mock community sampled. The AD, a metric specific for compositional data, was used as the distance metric to assess how close the expected abundances were to the observed. This work is unique as it provides an unbiased inclusion of publicly available mock communities, along with 5 samples specifically constructed for this analysis, an overview of the five shotgun metagenomics pipelines and downstream analysis considerations, three different accuracy metrics for assessment and a confusion matrix for the 5 constructed set of mock community samples specific to this study. Overall, we found bioBakery4 to have the closest taxonomic classification performance, followed by the Kraken2 based tools, JAMS and WGSA2. In contrast, the Kraken2 tools were more highly sensitive, but provided a greater number of false positives. To the best of our knowledge, at the time of submitting this work, there have been no studies comparing the performances between bioBakery4, JAMS, WGSA2, or Woltka.

### Ensuring unambiguous species identity

Bacteria names, especially down to the species and the strain level, can vary widely based on the bioinformatics pipeline, the chosen classifier, the database used and or the release date of any of these. Often taxonomy names frequently include extra identifiers on the scientific names themselves, especially down to the strain creating issues with name matching across data sets logistically challenging. In addition, organisms are constantly being reclassified, names may not be updated at the time of data inspection, or even outdated names may still be used which creates concordance issues with resolving names between data sets. The need to have a standardized a unique identifier that could be applied to all data sets, such as NCBI’s Taxonomy Identifiers (TAXID), was imperative to accurately assess performances across and between the pipelines used in this study. To address these issues, this work included the development of parsing and annotating the outputs of the included pipelines with TAXIDs so that proper comparison could be conducted. Future work would involve publishing the TAXID workflow included in this manuscript as a standalone Python package.

### Accuracy and performance

When a researcher is considering how to process and analyze their final relative abundance tables from their clinical samples, a key question addressed is how to and at what threshold to filter spurious bacteria very low abundances across their samples. Filtering of organisms below a certain threshold is a common approach in metagenomics, though there is no best filtering threshold in all cases^43^ and often times, filtering is done in an arbitrary way. Some researchers have addressed this such as in the Portik *et al*, publication where they utilized a 0.001%, 0.1%, and 1% (“mild”, “moderate”, “heavy”) type of filtering^41^. Whereas other authors, such as, Parks *et al*. simply specified a 0.01% filtering threshold^50^, and the MetaPhlAn4 authors utilize a 0.01% filtering threshold for Bracken^51^ results^13^. When no filtering is applied, one can consider those spuriously reported bacteria to be false positives or artifacts of the sequencing or potential artifacts of the bioinformatics pipelines used^41,50^. As a result, we concur with the previous literature and utilize a 0.01% RA filtering threshold.

In the 2017 publication by Gloor, he proposed the use of the AD for compositional data^47^. While in previous years, other distance metrics were more widely used, more recently other microbiome studies are using the AD for beta diversity measurements^52–55^. We used the AD in this work to assess the closeness of the overall observed compositional structure to the expected compositional structure for each mock community sample, and comparison of pipelines by taxonomic accuracy revealed several interesting trends. As shown in Table 2, the AD metrics showed that the bioBakery pipelines had the lowest overall AD values (best), with JAMS showing second best, followed by WGSA2 and then finally Woltka with the highest average of 24.51. Despite this trend using AD, the overall sensitivity metric did not follow this same ordering. Since sensitivity is calculated as a “hit” at any RA, closeness to the expected RA is not necessary. Additionally, sensitivity is not penalized for false positives, so a pipeline with many false positives could have not only a high sensitivity but also a high AD. Overall, there was an association between AD and FPRA since the AD is penalized for including extraneous species, but sensitivity followed a separate trend. These trends are explored below.

When considering the average AD over all samples, bioBakery4’s implementation of MetaPhlAn4 which utilizes MAGs^8,10^, boasts the lowest AD of 7.50. When compared to bioBakery3’s MetaPhlAn3, MetaPhlAn4 seems to have greatly increased the accuracy by reducing AD from 10.49 to 7.50. Also interesting is that the two Kraken2-based pipelines—JAMS and WGSA2—perform similarly with average AD scores of 13.77 and 14.55 respectively, despite the lack of assembly in WGSA2’s classification. For these communities, this result suggests that assembly is not completely necessary for accurate annotation with Kraken2. This idea is supported by conclusions drawn by Tamames *et al*. who found strong similarities between raw read and assembly methods for Kraken2 performance^56^. Finally, Woltka seems to perform significantly worse than the others, often due to a large number of spurious organisms. In fact, Woltka had, on average, about 144 false positive species across all of the pipelines, ranging from 55–239 species (Supplementary Table S7. Comparisons done by the Woltka authors using Bracken^51^ against simulated, ground-truth communities showed better taxonomic classification performance than Woltka when comparing with Bray-Curtis similarity. However, once considering phylogenetic distance (a main goal by the Woltka authors) through weighted UniFrac, the distance between Woltka and Bracken decreases immensely^21^. Using a compositionally and phylogenetically-aware distance metric, like ratio UniFrac^57^, may reveal a different trend for distances and would serve as an avenue of exploration in future work.

While AD is a useful metric for assessing distance between samples, there are other confounding variables which makes this difficult between communities. The question of whether a longer read length might improve any of these scores was addressed by comparing the AD to the read lengths of each mock community. Interestingly, across the communities assessed, there was no significant correlations found between AD and average read length within any of the pipelines (Fig. 6). For these short read methods, it may be that the change in read length is not sufficient to significantly improve accuracy.

When comparing and contrasting the sensitivity metric across the pipelines, WGSA2 found the most number of expected species while the other pipelines differed by approximately 3%. The Kraken2-based approaches identified more expected species (92.34% for WGSA2 and 87.86% for JAMS) while bioBakery3 and bioBakery4’s gene-marker method identified 84.60% and 79.38%, respectively. Woltka’s OGU method identified 81.05%. Due to bioBakery’s marker gene approach, it will only report results with high confidence, leading to fewer organisms. On the other hand, k-mer methods will attempt to perform exact matching on a greater number of k-mers, leading to a greater number of organisms. Woltka’s method scores somewhere between bioBakery3 and bioBakery4.

As discussed, however, WGSA2 and JAMS had overall greater AD, suggesting that the Kraken2 approaches either give more spurious results, thus increasing the AD, or give relative abundances with greater error (also increasing AD). The average sensitivity across all of the pipelines were similar, ranging between 79.37%–92.34%. Regardless, there were no statistically significant differences in sensitivities between the pipelines which indicates that they all were able to find a similar number of expected species.

This work included a metric to assess false positive relative abundance even though differing filtering parameters were not a part of the analysis. The FPRAs give a value to spurious bacteria that were found from each pipeline. When considering the FPRA values for this work, a similar trend to the AD was observed. First, the gene marker methods (bioBakery3/4) provided the lowest average FPRA values (6.03% and 5.63%, respectively) while the Kraken2-based methods coming in second (JAMS: 7.59%, WGSA2: 6.24%). This is likely due to the fundamental differences between the marker gene approach versus the k-mer approach: bioBakery will only annotate if the marker genes are found, which yields very high confidence in the presence or absence of an organism. However, it only operates on a subset of the reads where these markers are found^58^. Kraken2, on the other hand, works with k-mers and attempts to annotate all of the sequences. In doing so, Kraken2 yields a much greater number of organisms. Woltka had the highest FPRA of 27.07%. Given that the Aitchison Distance metric takes into account spurious bacteria, then it follows that the total FPRA should correlate with the AD values.

Finally, the percent unclassified could only be determined for JAMS and WGSA2 by default. It is interesting to note that, while WGSA2 struggled to annotate the majority of BMock12 (28.53% unclassified), JAMS was able to do so with only 1.32% unclassified.

Finally, a trend emerges among the samples tested as an average was calculated not only on a per-pipeline basis, but also on a per-community basis. The average of performance across each mock community can be adequately surmised as a “difficulty” metric for the community. In the communities sampled here, BMock12’s difficulty score is much higher than any other community. In AD, BMock12 is 31.80 on average while the next highest, NIST-MIX-C is 16.33. This trend applies to the other metrics as well (40% average sensitivity and 23.98% average FRPA) and is likely due to the difference in organism source, since the communities included in this study were mostly comprised of bacteria representative in the gut microbiome. The one exception was the BMock12 community, which was the most challenging community to profile because it was an environmental sample containing organisms from both hot and cold lakes. This community was constructed to also contain organisms with very low abundance including *Micromonospora coxensis* and *Micromonospora echinaurantiaca* with relative abundances of 0.00448% and 0.877%, respectively. Despite BMock12 being extremely deeply sequenced and having above-average read length, it still proved to be the most problematic for the pipelines as shown in Table 2. When considering all metrics assessing accuracy, BMock12 had the highest average AD (31.80), lowest average sensitivity (40.00%), and second highest FPRA (23.98%). This suggests that current databases have a dearth of these extreme environmental organisms. Researchers studying exotic environmental samples should be cautious to ensure pipelines are capable of detecting species of interest.

When considering the time performance of the pipelines, bioBakery4 performed the best with a time of 1:09 minutes per CPU, with JAMS and WGSA2 following closely behind at 1:33 and 1:43 minutes per CPU. This close grouping suggests relatively similar performance in terms of time complexity for bioBakery4, JAMS, and WGSA2. Considering the taxonomic performance of each pipeline, bioBakery4 performs the best and also has the fastest time. Separated from this group was bioBakery3 at a time of 7:23 minutes per CPU and Woltka with a time of 26:45 minutes per CPU. Woltka’s time consists mostly of the bowtie2 alignment step.

### Binary classification performance of NIST samples

Kralj *et al.*^59^ provided a framework for organism-by-organism assessment (see Confusion Matrix in Methods). A similar analysis on the 5 NIST mock community samples was included in this work, and this organism-centered approach revealed that Woltka and WGSA2 performed best when comparing F1 scores. Then, JAMS, bioBakery4, and bioBakery3 performed very closely in terms of F1 scores. However, the average specificity scores of WGSA2 and Woltka were among the lowest at 0% filtering (0.0 and 0.15), which were greatly improved to 0.77 and 0.69 at 0.01% filtering. This suggests that while highly sensitive, there may be issues with specificity when an organism is not present. This also underscores that filtering is an important factor to consider when performing the classification.

It is likely that due to misclassification of the infamous triad of *E. coli*, *Shigella spp*. and the closely related *Salmonella spp*.^60^, that almost all pipelines were unable to correctly identify *Shigella sonnei*. In fact, some *Shigella* and *E. coli* strains have nucleotide similarity of 99.9%^61^. Additionally, some *Salmonella* and *E. coli* strains have similarities of 98.6%^61^. This poses a difficult challenge for accurate differentiation between the highly similar species.

In these results, calculating the harmonic mean on non-missing values for each pipeline was extremely close (change in the thousandths place) to removing missing organisms from all confusion matrices. This suggests that calculation of the harmonic means can occur with the maximum number of features in each matrix, especially if the number of samples is low. Finally, recall that these accuracy metrics are binary classifications, that is, only the presence/absence is being tested rather than how close it is to the expected relative abundance. When evaluating overall agreement with expected mock community samples, AD is a better metric because it takes into account the distance between RAs.

### Other considerations in selecting a shotgun metagenomics pipeline

While downstream analyses were not benchmarked, it is worth discussing some of the downstream capabilities that each pipeline/package provides, as this may be considered when selecting a metagenomic pipeline. Consideration of which pipeline to use depends on the community of interest, the technical skill of the researcher, the questions posed, and what conclusions are desired. The work included here is an assessment of accuracy between an expected relative abundance and the observed relative abundance after taxonomic assignment, however each package provides other capabilities which may be important to researchers.

Microbial gene function potential is one of the overarching questions that researchers want to address when performing shotgun metagenomics sequencing. The bioBakery, JAMS, WGSA2, and Woltka pipelines all provide options for gene function assessment. For example, bioBakery3 and bioBakery4 includes bundled workflows comprised of many data processing options, including, HUMAnN2^23^, which provides profiling of the presence and/or absence of abundance of microbial pathways in a community including gene families, pathway abundances, and pathway coverage. In addition to these options, bioBakery4 can also perform MAG-based analysis. WGSA2, on the other hand, utilizes Prodigal^62^ for gene function prediction as part of the EggNogg-Mapper2^63^ for the Kyoto Encyclopedia of Genes and Genomes (KEGG) Orthology (KO)^64^, Enzyme Commission (EC), and Gene Ontology (GO)^65,66^ gene annotation. WGSA2 uses Minpath for pathway analysis (available at https://github.com/mgtools/MinPath). WGSA2 can also perform MAG analysis using MetaBat2 and scaffold analysis with Kraken2, which is useful for the detection of uncultured/unsequenced organisms in a community. Next, JAMS utilizes Prokka^67^ for gene annotation as well as InterProScan^68^ and can output these functional analyses as EC, GO, Pfam^69^, Product (from Prokka), Interproscan, or even antibiotic resistance genes. Currently, however, JAMS does not support microbial pathway analysis nor metagenome assembly. Finally, Woltka can provide functional analysis directly using its “coord-match” system, which matches reads to functional genes, and can output the results using functional catalogs such as UniRef, GO, KEGG, or MetaCyc.

Additionally, bioBakery, JAMS, and WGSA2 provide visualization utilities. For example, bioBakery has a visualization workflow which can generate box plots, heatmaps and ordination plots of quality control, taxonomic profiling, and functional profiling. WGSA2, as part of the default output, provides alpha diversity box plots and krona plots^70^ for visualization of taxonomy and pathway relative abundances. Additionally, WGSA2 provides the files in the BIOM format^71^, which can be directly imported into other programs (e.g., QIIME2^72^) for further analysis. Finally, JAMS includes a separate workflow for comparison between samples using any number of metadata variables called JAMSbeta, which can output highly customizable heatmaps and ordination plots across taxonomic or functional analyses. Moreover, JAMSbeta offers greater control over the outputs by allowing for customizing outputs directly within the R session using the data objects and plotting functions.

### Limitations

While this work has a multitude of strengths, such as an unbiased assessment of available tools for shotgun metagenomics processing, a method to robustly decipher taxonomy names across tools, and utilizing AD to assess closeness of compositional data, there are several limitations that should be mentioned. First, the five pipelines assessed in this work are only a small fraction of shotgun metagenomics processing tools that are available, therefore, this work does not attempt to provide a full assessment of every tool available but instead focused on more recent tools in the literature. To that note, this work purposefully benchmarked less popular tools in order to understand the different classification strategies from each of these less popular tools and compared these to a more commonly used tool such as bioBakery. Second, this work focuses primarily on assessing mock communities that mimic abundant/pathogenic gut microbiome organisms, so therefore the assessment here does not reflect any benchmarking work of representative oral, skin, vaginal environments. If the community of interest is not the human gut microbiome, these results may not accurately reflect the pipelines’ accuracy in those communities. Of course, the choice of these samples may not be reflective of all gut microbes, and using more diverse mock samples would be a future area of research. Moreover, the recommended database for each pipeline was utilized, and therefore a uniform reference database was not used across all of the pipelines evaluated. This may result in one reference database having a greater amount of reads as compared to another pipeline’s database, potentially biasing the accuracy results. As our aim was to focus on the accuracy of the tools, a comparison of different reference databases within each tool is outside of the scope of this analysis, but could be of benefit for future research if specific pipelines become more abundant in the microbiome literature. Another limitation is we were only able to determine the percent of unclassified reads for the WGSA2 and JAMS tools since bioBakery and Woltka work fundamentally differently and do not report unclassified by default. Furthermore, this work focused only on species-level data and therefore, higher taxonomic ranks may reveal different trends. Finally, the usage of the filtering threshold of 0.01% in this work was chosen arbitrarily. As in any clinically specific application, the filtering criteria used in the study could change the overall results of the report.

### Conclusions

Shotgun metagenomic studies for microbiome research are valuable, as they can be used to investigate the structural and functional properties of the microbial community and human health in association with the mechanisms of inflammation, immune response modulation, and metabolism, just to name a few^73–75^. Using shotgun metagenomics, researchers can obtain an understanding of the microbial community found within a sample at either the specificity up to the species level and sometimes strain level within the community. Since the human gut microbiome is composed of between 200-1000 species, interrogating and addressing the question of *who*, *what* and *how* much is present in the sample as accurately as possible is imperative^76^. Microbial composition of human host samples is not the only sampling niche of interest; there is also a large interest in identifying the microbial populations of environmental samples like soil and salt and fresh water environments^77^. Assessing bioinformatics tools, whether by generation of known communities or packages that perform statistical comparison, for shotgun metagenomic studies has become an area of increasing research and exploration (OPAL^78^, Grinder^79^, and FASTQSim^80^); this area of research is needed in order to elucidate the best tool for processing of microbial shotgun metagenomics sequence data. Since clinical trials are time consuming and costly, confidence in results for insights into interventions are critical. Depending on the research question, either high accuracy in taxonomic abundance or high sensitivity may be more important.

Overall, we find that bioBakery4 performs remarkably well with respect to taxonomic accuracy. However, in cases where sensitivity is absolutely critical, either JAMS or WGSA2 could be used. If limited in resources or CLI knowledge, then WGSA2 is preferred. Above all, we also reiterate the use of AD to compare the compositional closeness of data. Also, using samples with TP and TN mock communities is critical to evaluate tools on a granular, organism-centric perspective. Finally, we propose a way to annotate scientific names with TAXIDs.

## Methods

**Fig. 7:**
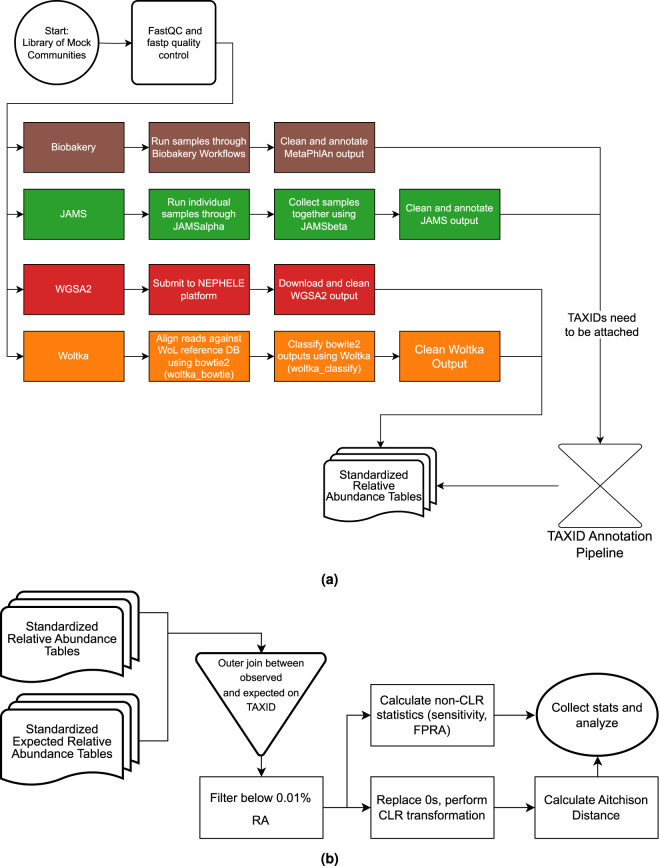
Flowcharts of the workflow for this study. (**a**) Flowchart showing the pipeline submission and standardization process for the benchmarking analysis. Colors indicate different pipelines. (**b**) Statstical evaluation of the the workflow for assessing the output from each pipeline. The name standardization pipeline is Fig. 1.

Figure 7a shows the overall workflow of this benchmarking report. Each mock sample was submitted to FastQC^81^ and fastp^82^ for quality control before submitting to each of the 4 respective pipelines. bioBakery3, bioBakery4, JAMS, and Woltka were processed on the NIH Biowulf supercomputing cluster (https://hpc.nih.gov/systems/). Once the resulting taxonomic count files from each pipeline were cleaned and were standardized, the relative abundance files were submitted to the next steps in the standardization process in order to assess the accuracy (Fig. 7b) of each pipeline for each mock sample assessed. Taxonomy files from bioBakery3, bioBakery4 and JAMS were submitted to the TAXID annotation pipeline developed for this project shown in Fig. 1 includes the workflow for naming standardization that was done from the NCBI Taxonomy ID files. The standardized names were then queried into the standardized NCBI taxonomy database to annotate them with TAXIDs.

### BMock12

The DNA mock community BMock12, from the One-to-One group, consists of twelve bacterial species/strains from 8 genera. This mock community was composed from a pool of separately extracted DNA, with the *Halomonas* strains grown in Hot Lake Heterotroph medium and the *Marinobacter* and *Psychrobacter* strains were derived from Antarctic Lake Vida^33,83^. See the original publication for the laboratory procedures. The FASTQ file generated from the Illumina HiSeq platform downloaded from NCBI SRA using accession SRR8073716^83^. Due to the massive sequencing depth (greater than 300 million reads), the sample was subsampled to 100.5 million reads using the seqtk toolkit https://github.com/lh3/seqtk with the following command (the same was run on the reverse read):


seqtk sample -s123 SRR8073716_1.FASTQ 100500000 \ > sub_SRR8073716_1.FASTQ


Supplementary Table S1a illustrates the expected relative abundances for this mock community.

### CAMISIM

The CAMISIM mock community, also from the One-to-One group, is a simulated mock community from the second CAMI challenge generated from the Critical Assessment of Metagenome Interpretation (CAMI) initiative. This initiative focuses on improving and assessing assembly, profiling, and binning methods^38^. The initiative included a tool called CAMISIM and its capabilities include simulation of complex metagenomic samples. The tool was used to develop the mock community datasets for the first CAMI challenge^25^. Using this tool, the authors also developed another test suite for the second CAMI challenge based on the HMP dataset^84,85^. Sample 1 of CAMISIM, selected from the HMP CAMI dataset, included 38 unique species composed from 17 genera and Sample 2 included 21 species composed from 9 genera^84^. The expected relative abundance tables from both CAMISIM samples are shown in Supplementary Table S1b and S1c.

### NIST

The 5 mock community samples provided by NIST belong to the One-to-One group since each mock sample was sequenced once and had different community structures. The expected RA tables for the five mock community samples provided from NIST are shown in Supplementary Table S1d. The NIST samples of pathogenic bacterial DNA mixtures were generated using NIST RM8376 Microbial Pathogen DNA Standards for Detection and Identification. Each DNA material was prepared as per the RM8376 handling instructions^34^. Five mixtures were generated in total by preparing 5 different pools of 2-3 species. The list of strains used and pools are also in Supplementary Table S1d; strains were combined in each pool to obtain similar genome copies for each strain in the final pool. Genome copies/ in the pool are listed in the final column. For the equigenomic mixture pools were added 1:1:1:1. For the remaining mixtures NIST-A–D, samples were combined in a Latin square design with pools being added over 4 log dilutions (undiluted to $1{0}^{-3}$). Pool E served as an internal control and was added to all pools at a 100-fold dilution. For mixes A–D, one pool was left out in each mixture, so the EG mixture was comprised of 14 bacterial pathogens total, and all other mixtures were comprised of 11 strains total. Once the mixtures had been generated, samples were prepared for shotgun sequencing using the NEBNext Ultra II FS DNA Library Prep Kit for Illumina (Cat # E7805S/L) with the following protocol for DNA inputs > 100ng with NEBNext Multiplex Oligos for Illumina Unique Dual Indexes (Cat # E6442S/L). DNA libraries were normalized and a 12pM library was loaded on the Illumina MiSeq platform to generate 2 x 150 bp reads (300-cycle MiSeq Reagent Kit v2, Cat # MS-102-2002). Raw FASTQ files were transferred using NIH BOX data repository and have since been published on SRA at Study Accession SRP436666^86^.

Since this study focuses on species-level analysis, *Salmonella enterica subsp. enterica* (subspecies-level, TAXID 59201) was reclassified as *Salmonella enterica* (species-level, TAXID 28901), and the serotype *Escherichia coli O157:H7* (serotype-level, TAXID 83334) was reclassified as *Escherichia coli* (species-level, TAXID 562).

### Amos HiLo and Mixed

The DNA mock community developed at the National Institute for Biological Standards and Control (NIBSC) by Amos *et al*. consisted of 20 common gut microbes, that were pure strains, and the extracted DNA was pooled together for both even and staggered mixtures. The mock samples from Amos *et al*. are from the “Replicates” group since each staggered and even community were sampled and sequenced 5 times. The staggered community is referred to as “HiLo” and the even community is referred to as “Mixed”. The 20 microbes in each sample spanned 19 species and were comprised of 16 genera^32^. The strain *Bifidobacterium longum subsp. longum* was renamed and combined with *Bifidiobacterium longum* for a total RA of 37% in the staggered community and 13% in the even community (reflected in Supplementary Table S1e). The 10 FASTQ files (five from Mixed and five from HiLo) generated from the NextSeq 500 platform were downloaded from NCBI SRA using accessions SRR11487931–SRR11487935^87^ for the HiLo community and accessions SRR11487937–SRR11487941^87^. Supplementary Table S1e illustrates the expected RA for this mock community.

### Tourlousse

The mock community developed by Tourlousse *et al*. consists of a near even mixture (average $5.26\pm 1.52 \%$) of 20 species composing 19 genera, according to the authors, developed to be control reagents for human gut microbiota. The authors developed both a DNA mixture and a whole-cell mixture however, only the DNA mixture was used for benchmarking in this report^31^. While the DNA community was replicated 20 times by various labs, six replicates from the same lab were selected for this study and therefore this set belongs to the ‘Replicates’ group. The strain *Bifidobacterium longum subsp. longum* was renamed and combined with *Bifidiobacterium longum* for a total relative abundance of 10.4%. The raw FASTQ files were downloaded from the NSCBI SRA using accession numbers SRR17380241–SRR17380246^88^. The expected relative abundances for each organism are summarized in Supplementary Table S1f.

#### Overview of workflows

The overall workflow for this project is described in Fig. 7. All computations were performed using the NIH High Performance Computing (HPC) Biowulf cluster (http://hpc.nih.gov). Briefly, all FASTQ sequences were first assessed for quality using FastQC and then trimmed to improve quality with fastp. Trimmed and quality checked FASTQ files were submitted through the 4 shotgun metagenomics pipelines/packages, typically with default parameters. The raw outputs from the pipelines were cleaned with custom Python scripts (d) and were further submitted to an NCBI Taxonomy ID (TAXID) annotation pipeline. This process converted the raw outputs into RA tables with standardized TAXIDs (Fig. 7a). Finally, all cleaned and standardized tables were collected and filtered for further analysis. Accuracy metrics and statistics were calculated against the known compositions. All figures of bar charts, heatmaps and bivariate plots were processed in custom Python scripts.

### Quality control and trimming

FASTQ files from each sample were quality control checked using FastQC (version 0.11.9)^81^. FastQC reports (not included) were collected using MultiQC (version 1.12)^89^ in order to visualize the initial quality. Upon inspection of quality, the tool fastp (version 0.23.2)^82^ was used for trimming and adapter removal of all samples—except Amos Mixed and Amos HiLo—with the following parameters: q=15, cut_front, and cut_tail. For the Amos Mixed and Amos HiLo datasets, the following were used: q=25, cut_front, cut_tail, l=100, f=15, t=75.

#### Resolving taxonomic species names across pipelines with NCBI taxonomy Identifiers

Results from bioBakery and JAMS do not include NCBI taxonomy identifiers (TAXIDs) and therefore the relative abundance tables from these two packages were submitted to the taxonomy annotation pipeline. The pipeline begins with the NCBI Taxonomy database which downloaded from the publicly available FTP server (https://ftp.ncbi.nih.gov/pub/taxonomy/) on Feb 13, 2023. The NCBI taxonomy names were then standardized to allow for matching across different pipelines. The genus name (first word) and species names (including strain tags) were split into two separate strings. Non-alphabetical characters in the species names or afterward were replaced with a “-". If there was a strain identifier, it was attached to the species name with a “-". Next, any “[“ or “]" characters were removed from tentative genera. Finally, everything was concatenated together with a “_" and capitalized. Since there are also higher-ranked taxonomic names consisting of one word, such as the genus *Escherichia*, these were only capitalized since further standardization was not necessary. This yielded the NCBI standardized database specific to this report, which was used for querying of TAXIDs, and this was saved as a .pkl file. This process is summarized in Fig. 1. As an example, from BMock12, *Marinobacter sp. LV10R510-8* would be converted into “MARINOBACTER_SP._LV10R510-8".

The next step in this pipeline was to annotate all organisms with a TAXID. Raw organisms names from bioBakery and JAMS were standardized as described above. Then, the NCBI .pkl file was loaded and the standardized name was queried, which yielded the corresponding TAXID. If any names could not be annotated, an error was raised with the offending names.

Out of all of the pipeline outputs, only the following edits were made to species names before the standardization step. The output from bioBakery3 and bioBakery4 included name editing for two mock sets, the Amos samples and the Tourlousse samples. The species “Prevotella_copri_clade_A” was renamed to “Prevotella_copri” and the species “Clostridium_clostridioforme” was renamed to “Clostridium_clostridiiforme”. The JAMS output required no name editing standardization. Also, since the output from both WGSA2 and Woltka included TAXIDs, there was no need to perform any species-TAXID linking on these two pipelines.

Using the name standardization process allowed for accurate comparison of samples, even if naming diverged. After running on the JAMS and bioBakery samples, all the annotated relative abundance tables were completed with the standardized names and TAXID identifiers and ready for analysis.

#### Generation of relative abundance tables by four pipelines

The RA tables (format of Species Name, Relative Abundance, TAXID) for the pipelines were generated in several steps. First, the raw output was cleaned to gather the species/genus level data and their RA without TAXIDs (un-annotated). Then, using NCBI’s taxonomy database, the names were standardized and TAXIDs were assigned to each organism to permit accurate comparison, even if the names differed. This process yielded the annotated relative abundance tables that were used in the analysis. False positive species with extremely low relative abundance values (<0.01%) were removed from all tables before any metrics were calculated.

### bioBakery Workflows

The bioBakery Workflows package, developed by the Huttenhower lab, (https://github.com/Biobakery/Biobakery/wiki/Biobakery_workflows) used in this work, included two versions of bioBakery4 Workflows: 3.0.0-alpha.7, utilizing MetaPhlAn3 (version 3.0.14) and 3.1, utilizing MetaPhlAn4 (version 4.0.4). The outputs from the two versions will be referred to as bioBakery3 and bioBakery4. While the bioBakery workflow is a package, the results of this work focuses only on the output from the MetaPhAn profilers from within the packages. While both versions of MetaPhlAn utilizes clade-specific marker genes, MetaPhlAn4 also integrates metagenomic assembled genomes (MAGs). The classification database of MetaPhlAn3 marker genes were derived from a dataset of microbial reference genomes using UniProt derivatives and the ChocoPhlAn 3 pipeline^23^ to produce genomes and gene families used for taxonomic and functional annotation, The classification database of species-level genome bin-specific markers in MetaPhlAn4 was built using replicated and quality-controlled genomes in several steps^13^ to produce both known and unknown species-level genome bins. The exact command that was executed was the following


biobakery_workflows wmgx --input-extension fastq --input $INPUT --output $OUTPUT \ --threads 32


Both versions utilized kneaddata v0.12.0 (https://github.com/biobakery/kneaddata) for decontamination, quality control, and trimming as part of the workflow. The FASTQ files from the mock communities were submitted to biobakery_workflows using the “wmgx” flag for whole genome shotgun metagenomics.

Species level relative abundances were extracted from bioBakery4’s merged MetaPhlAn3 and MetaPhlAn4 output with the following command:


grep -E "(s__)|(taxonomy)" metaphlan_taxonomic_profiles.tsv \ > "species_relab.txt"


Species-level relative abundances from the MetaPhlAn4 outputs, were extracted with the following command since the output included MAG classifications (the “t__” tag needed to be removed):


grep -E "(s__)|(taxonomy)" metaphlan_taxonomic_profiles.tsv | grep -v "t__*" \ > species_relab.txt


The above commands yielded the species-level RA without TAXID annotations. These intermediates were then annotated with NCBI TAXIDs, yielding the final standardized relative abundance tables.

### Just A Microbiology System (JAMS)

JAMS is a metagenomic workflow developed by John McCulloch at the National Cancer Institute and is available at https://github.com/johnmcculloch/JAMS_BW^17^. JAMS is divided into two main pipelines, JAMSalpha and JAMSbeta. The JAMSalpha pipeline runs the processing of the FASTQ files and performs taxonomy. JAMSalpha utilizes trimmomatic^90^ for preprocessing/trimming, Kraken2 for decontamination, megahit^91^ (v1.2.9) for assembly and Kraken2 for annotation. The database used for Kraken2 is a curated database created by the JAMS authors. The database is a formatted database which contains compiled bowtie2 indices of all complete genomes from *Homo sapiens* and *Mus musculus*, Bacteria, Archaea, Fungi, Viruses, and Protozoa from NCBI taxonomy. The database is created using a script within the JAMS pipeline. The database is available for download and public use on the section 2 of the JAMS alpha documentation wiki here: https://github.com/johnmcculloch/JAMS_BW/wiki/JAMSalpha. The source code used for building the database is part of the JAMS package and can be found here https://github.com/johnmcculloch/JAMS_BW/blob/master/libexec/JAMSbuildk2db. It was last created December 2022 from the entire NCBI database and the version used was JAMSdb202201_1.6.6_20220114.

JAMSbeta is a post-processing pipeline and is used for analysis once taxonomy is assigned. JAMSbeta can perform between sample analyses with provided metadata and can perform analyses of *α* or *β* diversity metrics from relative abundances.

This report used version JAMS v1.7.9. For the purpose of this report, JAMSalpha was run creating a swarm file for each community. To do so, the following command was used (leveraging the bundled JAMSmakeswarm utility):


JAMSmakeswarm -r [path/to/reads] -d [path/to/db]


In the SLURM swarmfile, each sample had the following command executed on it once the swarmfile was submitted for calculation:


JAMSalpha -f [path/to/f_read] -r [path/to/r_read] -o [path/to/output] \ -H human -p [prefix]


Once the JAMSalpha jobs completed, JAMSbeta was run in order to generate the relative abundance table. To do so, the following command was used:


JAMSbeta -p [projectname] -o [output] -t [metadata].tsv -y [path/to/jamsfiles] \ -e -k -z -n LKT,ECNumber,Product,Pfam,Interpro,GO,resfinder


The output of JAMSbeta yielded an Excel file with the relative abundance of each sample. Using another custom Python script and openpyxl—a Python package which can parse Excel files—the species were extracted from the Excel file and stored in an intermediate unannotated relative abundance table. This table was processed through the TAXID annotation pipeline for the resolution of TAXIDs. The “Unclassified" row was also kept in the tables. Furthermore, the unclassified row also contained organisms which were assigned at higher taxonomic ranks, as the output lineage gave them as unclassified at these lower ranks.

### Whole MetaGenome Sequence Assembly pipeline (WGSA2)

WGSA2 is a metagenomic workflow developed by Angelina Angelova, as part of the Nephele cloud-based microbiome analysis tool^16^. This pipeline utilizes fastp for trimming and error-correction, then Kraken2 for decontamination. Then, the trimmed, error-corrected, and decontaminated reads (TEDreads) are annotated with Kraken2, yielding the relative abundance taxonomic tables which were used in this study. Specifically, the Kraken2 database used in the pipeline was custom-built on Nov 8, 2021 and details can be found on the wiki at https://nephele.niaid.nih.gov/details_wgsa/. WGSA2 is also capable of scaffold-based analysis, gene-based functional analysis, and MAG-based taxonomic abundances, though these require assembly and were not used.

WGSA2 must be run on the Nephele platform (https://nephele.niaid.nih.gov/). All mock community samples were uploaded from the Biowulf cluster using the file transfer tool Globus (https://www.globus.org/). Metadata files for each community were created and required for each upload. All file uploads were submitted separately by mock community and the parameters used can be found in Supplementary Table S3. The results of a run are provided as a zip file for download.

For each WGSA2 Nephele run, the relative abundance tables were taken from the TAXprofiles/TEDreadsTAX/reports directory. The relative abundance was determined by dividing the counts for the organism by the total number of counts. Additionally, WGSA2 includes a TAXID annotation for each organism, thus, TAXID annotation pipeline was not needed.

### Woltka

Woltka is a taxonomic classifier developed by Zhu *et al*., and utilizes a fundamentally different classification scheme than the other classifiers included here^21^. It is based on operational genomic units, a similar idea to the 16s Operational Taxonomic Unit classification scheme^92,93^. Woltka is also phylogeny-aware, taking into account the evolutionary distances between organisms, which facilitates metrics such as weighted UniFrac. Importantly, Woltka is not a full pipeline, but rather only a classifier. Quality control and alignment must be performed before taxonomic classification.

First, the reads must be aligned using bowtie2^94^ against the Web of Life (WoL) database (http://ftp.microbio.me/pub/wol-20April2021/) using the following command, referred to as the “SHOGUN” protocol by the Woltka authors:


bowtie2 -p 16 -x [path/to/WoLdb] -1 [f_read] -2 [r_read] \ --very-sensitive --no-head --no-unal -k 16 --np 1 --mp "1,1" \ --rdg "0,1" --rfg "0,1" --score-min "L,0,-0.05" \ | cut -f1-9 | sed "s/$/\t*\t*/" | gzip > [sampleID].sam.gz


The SAM file output was submitted for taxonomic classification using following command:


woltka classify -i [sam_file] --to-tsv -o [output_dir_name] \ -r genus,species --map [WoLdb/taxid.map] --nodes [WoLdb/nodes.dmp] \ --names [WoLdb/names.dmp]


For Woltka, the output directory contains two files: genus.tsv and species.tsv. The result files include three columns: TAXID, counts, organism name. Relative abundances were calculated directly from the count column by dividing the count of each organism by the total sum of counts. Additionally, the TAXIDs were immediately available and therefore the TAXID annotation pipeline was not needed.

#### Binary classification using confusion matrices on the NIST samples

Kralj *et al*. propose a paradigm of *organism-centric* analysis for assessment of binary classification performance metrics, such as F1 scores^59^. Briefly, F1 scores are a way of assessing binary classification performance on both precision and sensitivity (recall). Since it is the harmonic mean, it takes into account the performance in both metrics. A score of 1 would imply perfect precision and sensitivity. Therefore, following this methodology, confusion matrices and performance metrics were generated for the five NIST samples, since the presence/absence of the same organisms across five separate samples was known. First, the standardized relative abundance tables from the expected and all of the pipelines were left joined. Then, for each pipeline, a separate pivoted dataframe was created which allowed for easy comparison by species across the five samples. Using the pivoted dataframe, the observed relative abundances were compared with the expected in each sample. Four conditions were then possible: true positive (FP), false positive (FP), false negative (FN), and true negative (TN). Additionally, two filtering thresholds were chosen (0% and 0.01%). A result was considered a TP if both the expected and observed were above the threshold; a FP if the expected value was less than or equal to the threshold, but the observed was greater; a FN if the expected value was greater than the threshold but the observed was less than or equal to the threshold; and finally a TN if the expected was equal to or less than the threshold and the observed was also less than or equal to the threshold. Fortunately, none of the expected species were below the 0.01% filtering threshold, which would have caused ambiguity in TP vs FN. Also, pipelines which did not report the species (missing values) were assigned RA of 0% in the observed.

In effect, the 0% threshold means that a TP would be detected if both the observed and expected had more than 0% RA. A FN or TN could only occur at 0% filtering if the pipeline either did not report the species (assigned 0%) or actually reported it as 0%.

For performance metrics (PMs), we used the following definitions of sensitivity, precision, specificity, accuracy and F1 score.

1 $$Sens=\frac{TP}{TP+FN}$$

2 $$Prec=\frac{TP}{TP+FP}$$

3 $$Spec=\frac{TN}{TN+FP}$$

4 $$Acc=\frac{TP+TN}{TP+TN+FP+FN}$$

5 $$F1=\frac{2\cdot Sens\cdot Prec}{Sens+Prec}$$

The harmonic mean and arithmetic mean were used to assess the performance of each pipelines classification performance from the confusion matrix. While the harmonic mean is the recommended operation by Kralj *et al*., the arithmetic mean was calculated as a point of comparison. Unfortunately, the performance metrics could not always be calculated due to missing values, which also meant that the summary statistics could not be calculated. To combat this, the harmonic mean was calculated by ignoring any missing values. This method was validated by removing an organism from all matrices if any pipeline had a missing F1 score, and changes to F1 scores were only perturbed in the thousandths place. A summary of F1 scores is given in Table 5a, b. A graphical representation of the data can be visualized in Supplementary Figure S1 and S2.

**Table 5:** Tables of aggregated F1 scores from confusion matrix analysis.

F1 Score at 0.0% Threshold					
Species	bioBakery3	bioBakery4	JAMS	WGSA2	Woltka
*Achromobacter xylosoxidans*	0.857	0.857	0.857	0.889	0.889
*Acinetobacter baumannii*	0.857	0.857	0.857	0.889	0.889
*Aeromonas hydrophila*	0.889	0.889	0.889	1.000	1.000
*Enterococcus faecalis*	1.000	1.000	0.857	0.889	0.889
*Escherichia coli*	0.889	0.889	0.889	0.889	0.889
*Klebsiella pneumoniae*	0.857	0.857	1.000	0.889	0.889
*Legionella pneumophila*	1.000	1.000	1.000	1.000	1.000
*Listeria monocytogenes*	0.857	0.857	0.857	0.889	0.889
*Neisseria meningitidis*	0.857	0.857	0.857	0.889	0.889
*Salmonella enterica*	0.857	0.857	0.889	0.889	0.889
*Shigella sonnei*	—	—	1.000	0.889	0.889
*Staphylococcus aureus*	0.857	0.857	0.857	0.889	0.889
*Streptococcus pyogenes*	0.857	0.857	0.857	0.889	0.889
*Vibrio furnissii*	0.857	0.857	0.857	0.889	0.889
**Harmonic Mean**	0.881	0.881	0.891	0.903	0.903
**Mean**	0.884	0.884	0.894	0.905	0.905
**a Summarized table of F1 scores from confusion matrix analysis by pipeline at 0.01% filtering threshold**.					
F1 Score at 0.01% Threshold					
Species	bioBakery3	bioBakery4	JAMS	WGSA2	Woltka
*Achromobacter xylosoxidans*	0.857	0.857	0.857	1.000	1.000
*Acinetobacter baumannii*	0.857	0.857	0.857	1.000	1.000
*Aeromonas hydrophila*	0.889	0.889	—	1.000	1.000
*Enterococcus faecalis*	0.857	1.000	0.857	1.000	1.000
*Escherichia coli*	0.889	0.889	0.889	0.889	0.889
*Klebsiella pneumoniae*	0.857	0.857	0.857	1.000	0.889
*Legionella pneumophila*	1.000	1.000	0.889	1.000	1.000
*Listeria monocytogenes*	0.857	0.857	0.857	1.000	1.000
*Neisseria meningitidis*	0.857	0.857	0.857	1.000	1.000
*Salmonella enterica*	0.857	0.750	0.889	0.889	0.889
*Shigella sonnei*	—	—	0.857	—	—
*Staphylococcus aureus*	0.857	0.857	0.857	0.857	1.000
*Streptococcus pyogenes*	0.857	0.857	0.857	1.000	0.857
*Vibrio furnissii*	0.857	1.000	0.857	1.000	—
**Harmonic Mean**	0.872	0.881	0.864	0.969	0.957
**Mean**	0.873	0.886	0.864	0.972	0.96
**b Summarized table of F1 scores from confusion matrix analysis by pipeline at 0.01% filtering threshold**.					

Individual stratified subtables can be found in Supplementary Table S4 and S5.

## Accuracy Metrics and Statistical Analysis

### Aitchison Distance

Many common distance or dissimilarity metrics (e.g., UniFrac^95^, Jaccard^96^, and Bray-Curtis^97^) fail to take into account the compositional nature of microbiome experiments. An analysis by Gloor *et al*. describes the problems which are precipitated by using non-compositional distance approaches, such as unconstrained correlation and covariation, as well as a non-linear relationship in Euclidean space^47^. To combat this, Gloor advises the use of log-ratios in the methods prescribed by Aitchison^98,99^. To this note, this report uses the Aitchison distance (AD) metric to assess closeness or proximity of the observed relative abundance values to the expected relative abundance values. Briefly, first the geometric mean (*G*(*x*)) of the sample is obtained, where *x*_*D*_ represents a feature in a vector of length *D*. It is important to note that this operation can be done on feature counts or relative abundances and will still yield the same CLR-transformation.

6 $$G(x)=\sqrt[D]{{x}_{1}\cdot {x}_{2}\cdot \ldots \cdot {x}_{d}}$$

Then, each feature is divided by the geometric mean, and the logarithm is taken:

7 $${x}_{clr}=\left[log\frac{{x}_{1}}{G(x)},log\frac{{x}_{2}}{G(x)},\ldots ,log\frac{{x}_{D}}{G(x)}\right]$$

This yields the new CLR-transformed vector. The AD is the Euclidean distance of the CLR values. For example, given CLR-transformed vectors $x{\prime}$ and $y{\prime}$, both of length *D*, then the Aitchison Distance is the familiar Euclidean distance equation:

8 $$AD=\sqrt{{(x{{\prime} }_{1}-y{{\prime} }_{1})}^{2}+{(x{{\prime} }_{2}-y{{\prime} }_{2})}^{2}+\ldots +{(x{{\prime} }_{d}-y{{\prime} }_{d})}^{2}}$$

However, there are problems with this approach. Notably, zero-valued features are intractable as the logarithm of zero is undefined. There are several approaches to solving this problem, many of which are explored by Lubbe, Filzmoser, and Templ^100^. The present study uses the multiplicative replacement method, originally devised by Fernández, to replace zeroes in the relative abundance vectors^101^. The particular implementation was provided by the scikit-bio package (available at https://github.com/biocore/scikit-bio). After replacing the zeroes, the steps in Eqs. (6), (7), and (8) are applied to the vector to calculate the Aitchison Distance.

### Total False Positive Relative Abundance

The FPRA is calculated with the same procedure as Amos *et al.*^32^. The total FPRA is the total sum of all false positive relative abundances.

9 $${\rm{FPRA}}=\frac{{\rm{Abundance}}\,{\rm{of}}\,{\rm{False}}\,{\rm{Positive}}\,{\rm{Species}}}{{\rm{Total}}\,{\rm{Abundance}}}\times 100 \% $$

To calculate FPRA, rows were selected where the expected RA was 0%. The sum of the observed RA from these rows, divided by the sum of the observed RA, yielded the FPRA. This metric provides a check to Sensitivity (Eq. (10), below) as finding all of the species does not mean it did so without spurious results. Also important to note is that overestimation of relative abundances did not count toward FPRA. For example, if a pipeline were to indicate a sample consisted 100% of an expected species, the FPRA would still be 0%. Additionally, for pipelines which gave the percentage unclassified, these were not considered false positives. That is, only annotated species were considered a false positive.

### Sensitivity

The sensitivity is a metric that provides the percent of total true positive species found that were expected.

10 $${\rm{Sensitivity}}=\frac{{\rm{Number}}\,{\rm{of}}\,{\rm{Correctly}}\,{\rm{Identified}}\,{\rm{Species}}}{{\rm{Total}}\,{\rm{Number}}\,{\rm{of}}\,{\rm{Expected}}\,{\rm{Species}}}\times 100 \% $$

Calculation was performed by counting the number of rows where both the expected and observed RA were greater than zero (corresponding to a hit), then dividing by the number of expected species. This metric allows for determination of how well a pipeline can find the expected organisms in a community.

### Unclassified percentages

The percent of unclassified reads of each sample was given by JAMS and WGSA. The WGSA simply gave the percentage in the output file as the first line labelled “Unclassified”. For JAMS, there was also an “unclassified” row which was used. However, organisms which were classified at a higher rank were also considered “Unclassified” by the JAMS pipeline, and these were added together for the total percent unclassified. For the replicates, these percentages were averaged together.

### Statistical analysis

The average accuracy metric over all samples for each pipeline was calculated and denoted the pipeline’s “Average Score” in Tables 2, 3, and 4. To compare average scores, first a Kruskal-Wallis test was conducted to see if any significant differences existed across the groups’ medians (implemented by the scipy.stats package)^102^. Then, a pairwise, post-hoc Wilcoxon test^103^ was conducted to see which pipelines had significant differences (also implemented by the scipy.stats package). Since multiple comparisons were conducted, the Benjamini-Hochberg FDR procedure^104^ was applied to the p-values (implemented by the statsmodels package), and the corrected values are tabulated in Supplementary Table S2a–c. Data in the manuscript are presented as *μ* ± *SD*.

#### Time and Memory Performance

The time performance metrics were generated from the NIST samples (i.e., EG, A, B, C, D). To do this for each pipeline a separate set of steps was followed. First, for bioBakery3 and bioBakery4, the AnADAMA2 log output was read and parsed for the starting time (*t*_1_), completion time (*t*_2_), and number of CPUs (*n*). The final time per CPU was calculated with the following equation:

11 $$\frac{{t}_{2}-{t}_{1}}{n}$$

Next, for JAMS, the JAMS.log files were similarly parsed for the starting time, ending time, and number of CPUs. However, since JAMS runs each job in a parallel fashion using the Biowulf-developed swarm utility (https://hpc.nih.gov/apps/swarm.html), the average time elapsed per job was then divided by the number of CPUs. The number of CPUs used per job was constant. For WGSA2, the WGSA logfile.txt was parsed for a starting time, end time, and number of CPUs. Then, Eq. (11) was used to calculate the time per CPU. Finally, for Woltka, the runs were separated into two stages. First, the bowtie2 alignment times were parsed from SLURM time logs and aggregated. Since the Woltka jobs were also run in parallel (using swarm), the time elapsed per job was averaged. Similarly, for the classification step, the elapsed times were parsed from the SLURM time logs, aggregated, and averaged together. The sum of the average bowtie2 alignment time and Woltka classification time was then divided by the number of CPUs to yield the average time per CPU.

For RAM, the specific usage depended on several factors. The bioBakery3 and bioBakery4 pipelines utilize an internal queuing system that can perform parallel work and scales on the amount actually needed, though when submitted, only one allocation of RAM was needed. Resource allocation depended on the size of the sample, but 128 GB was the most common allocation in this study. In contrast, the JAMS software package operates on a parallel processing framework. Each sample runs independently but in parallel. The authors advise 246 GB of RAM per sample, meaning a large amount of RAM could be used for multiple samples running in parallel. The WGSA2 platform was run through their online platform. Since it was not performed on local servers and provided as a service, the effective RAM utilization for the user is 0 GB. Lastly, the Woltka suite required two steps: the Bowtie2 alignment and then the classification step. The Bowtie2 alignment is resource-intensive, requiring in some cases 128 GB. Conversely, the classification step requires very little resources and usually around 16 GB were allocated.

### Supplementary information

Supplementary Information

## Data Availability

The mock microbiome community samples from NIST are deposited at the Sequence Read Archive at NCBI at Study Accession SRP436666^86^. The data used to generate the tables, figures, and heatmaps can be found within the GitHub repository. The raw data and code were also deposited to a figshare repository^105^. The raw output from the pipelines can be found in the utils/mock_communities.tar.gz archive. The cleaned and standardized outputs for both the expected and observed are found in the pipelines and expected_pipelines folders, respectively.

## Code availability

All relevant code used in these analyses can be found at https://github.com/mvee18/benchmarkingpaper and in the figshare repository^105^. The README in either repository provides additional useful information for the usage and description of files.
