# Supplementary material for: Mock community taxonomic classification performance of publicly available shotgun metagenomics pipelines
Source: Sci Data. 2024 Jan 17;11:81. doi: 10.1038/s41597-023-02877-7 (PMC10794705; doi:10.1038/s41597-023-02877-7)
Supplement: Supplementary file 1 — Supplementary Information [file 41597_2023_2877_MOESM1_ESM.pdf]

Supplementary Information

List of Tables

S1 Expected Relative Abundance tables for all communities. S4
S2 Pairwise Wilcoxon tests for AD, Sens, and FPRA. The FDR (Benjamini-Hochberg procedure) corrected p-values are shown in the table. Bold values indicate FDR-BH < 0.05. . . . . S5
S3 Table of WGSA2 parameters used. S5
S4 Confusion matrices for bioBakery3, bioBakery4, JAMS, WGSA2, and Woltka at 0% filtering threshold. tp = True Positive, fp = False Positive, fn = False Negative, TN = True Negative, sens = Sensitivity, spec = specificity, prec = Precision, acc = Accuracy, f1 = F1 Score S8
S5 Confusion matrices for bioBakery3, bioBakery4, JAMS, WGSA2, and Woltka at 0.1% filtering threshold. tp = True Positive, fp = False Positive, fn = False Negative, TN = True Negative, sens = Sensitivity, spec = specificity, prec = Precision, acc = Accuracy, f1 = F1 Score S11
S6 Relative Time per CPU for all NIST samples. If multiple samples were run in parallel, the average was taken. The Woltka time is the sum of both the alignment and classification steps for each sample. S11
S7 Table of the Number of False Positive species. A perfect score is 0. . . . . S11
S8 Table of Unclassified metrics. The values shown are percentages. A perfect score is 0%. Neither bioBakery nor Woltka give the number of unclassified by default. S12

List of Figures

S1 Bar Plot graphical representation of confusion matrices shown in Supplemental Table S4. . . . . S13
S2 Bar Plot graphical representation of confusion matrices shown in Supplemental Table S5. . . . . S14

Species	RA (%)	TAXID
Halomonas sp. HL-93	19.49	1666906
Muricauda sp. ES.050	18.03	1798204
Halomonas sp. HL-4	17.43	1761789
Marinobacter sp. LV10R510-8	15.75	1415571
Psychrobacter sp. LV10R520-6	11.11	1415574
Cohaesibacter sp. ES.047	7.07	1798205
Marinobacter sp. LV10MA510-1	4.88	1415567
Thioclava sp. ES.032	3.15	1798206
Propionibacteriaceae bacterium ES.041	1.16	1945889
Micromonospora echinofusca DSM 43913	1.04	47858
Micromonospora echinaurantiaca DSM 43904	0.88	47857
Micromonospora coxensis DSM 45161	0.00448	356852

(a) Expected relative abundance for BMock12.

Species	RA (%)	TAXID
Bordetella pertussis	25.41	520
Bordetella petrii	18.14	94624
Bordetella pseudohinzii	9.73	1331258
Clostridium aceticum	7.50	84022
Bacteroides thetaiotaomicron	6.64	818
Clostridium butyricum	3.24	1492
Bacteroides fragilis	3.10	817
Clostridium botulinum	2.98	1491
Clostridium pasteurianum	2.65	1501
Clostridium scatologenes	2.21	1548
Fermentimonas caenicola	1.33	1562970
Bacteroides salanitronis	1.33	376805
Bordetella bronchiseptica	1.33	518
Clostridium autoethanogenum	0.96	84023
Akkermansia muciniphila	0.89	239935
Bacteroides vulgatus	0.89	821
[Clostridium] stercorarium	0.89	1510
Clostridium acetobutylicum	0.89	1488
Escherichia coli	0.89	562
Eubacterium limosum	0.89	1736
Lachnoclostridium phytofermentans	0.81	66219
Clostridium saccharobutylicum	0.74	169679
Bordetella holmesii	0.70	35814
Achromobacter xylosoxidans	0.44	85698
Bacteroides dorei	0.44	357276
Erysipelothrix rhusiopathiae	0.44	1648
Burkholderia multivorans	0.44	87883
Clostridium carboxidivorans	0.44	217159
Bifidobacterium animalis	0.44	28025
Bordetella parapertussis	0.44	519
Ruminococcus albus	0.44	1264
Butyrivibrio hungatei	0.44	185008
Clostridium cellulovorans	0.44	1493
Odoribacter splanchnicus	0.44	28118
Bordetella bronchialis	0.44	463025
Clostridium tetani	0.36	1513
Clostridium beijerinckii	0.14	1520
Cellulosilyticum lentocellum	0.08	29360

(b) Expected relative abundance for CamiSim Sample 1.

Species	RA (%)	TAXID
Bordetella pertussis	36.65	520
Achromobacter xylosoxidans	14.74	85698
Clostridium botulinum	12.40	1491
Roseburia hominis	8.76	301301
Clostridium perfringens	8.51	1502
Bacteroides fragilis	3.76	817
Clostridium scatologenes	3.59	1548
Bacteroides thetaiotaomicron	2.39	818
Bacteroides dorei	1.82	357276
Clostridium cellulovorans	1.59	1493
Clostridium butyricum	1.26	1492
Ruminiclostridium thermocellum	0.80	1515
Clostridium aceticum	0.75	84022
Parabacteroides distasonis	0.55	823
Butyrivibrio hungatei	0.40	185008
Desulfovibrio africanus	0.40	873
Clostridium pasteurianum	0.40	1501
[Clostridium] stercorarium	0.40	1510
Clostridium saccharobutylicum	0.33	169679
Clostridium sporogenes	0.25	1509
Parabacteroides sp. YL27	0.25	1796646

(c) Expected relative abundance for CamiSim Sample 2.

Pool	Species	Copies ($\times 10^6$)	Volume to Add (uL)	EG (%)	Mix-A (%)	Mix-B (%)	Mix-C (%)	Mix-D (%)	TAXID
Pool A	*Escherichia coli*	884	100	8.80	30.40	0.04	0.00	2.76	83334
	Staphylococcus aureus	884	53.6	8.80	30.40	0.04	0.00	2.76	1280
	Neisseria meningitidis	884	40.8	8.80	30.40	0.04	0.00	2.76	487
Pool B	*Salmonella enterica*	768	79	7.60	2.60	30.10	0.03	0.00	28901
	Acinetobacter baumannii	768	63.9	7.60	2.60	30.10	0.03	0.00	470
	Klebsiella pneumoniae	768	100	7.60	2.60	30.10	0.03	0.00	573
Pool C	*Streptococcus pyogenes*	728	32.3	7.20	0.00	2.90	29.10	0.02	1314
	Achromobacter xylosoxidans	728	100	7.20	0.00	2.90	29.10	0.02	85698
	Shigella sonnei	728	75.3	7.20	0.00	2.90	29.10	0.02	624
Pool D	*Enterococcus faecalis*	970	65.8	9.60	0.03	0.00	3.90	30.00	1351
	Vibrio furnissii	970	100	9.60	0.03	0.00	3.90	30.00	29494
	Listeria monocytogenes	970	55.8	9.60	0.03	0.00	3.90	30.00	1639
Pool E (controls)	*Legionella pneumophila*	1363	100	0.14	0.47	0.53	0.54	0.43	446
	Aeromonas hydrophila	1363	71.2	0.14	0.47	0.53	0.54	0.43	644

(d) Expected relative abundances for the NIST mixtures. The final expected DNA concentrations for all members of the EG mixture is 40 $\mathrm{ng\,\mu L^{-1}}$. The final expected DNA concentration for all members of Mix A, B, C, and D is 11 $\mathrm{ng\,\mu L^{-1}}$.

Species	RA (%) HiLo	RA (%) Mixed	TAXID
Akkermansia muciniphila	0.18	6.37	239935
Alistipes finegoldii	1.30	4.54	214856
Anaerostipes hadrus	1.75	6.11	649756
Bacteroides thetaiotaomicron	7.72	2.69	818
Bacteroides uniformis	1.05	3.66	820
Bifidobacterium longum	37.02	12.92	216816
Blautia wexlerae	0.11	3.77	418240
Clostridium butyricum	10.59	3.70	1492
Collinsella aerofaciens	1.99	6.95	74426
Escherichia coli	9.33	3.26	562
Eubacterium hallii	1.48	5.16	39488
Faecalibacterium prausnitzii	0.16	5.49	853
Lactobacillus gasseri	0.26	8.97	1596
Parabacteroides distasonis	10.10	3.52	823
Prevotella copri	13.84	4.83	165179
Prevotella melaninogenica	1.53	5.34	28132
Roseburia hominis	1.35	4.72	301301
Roseburia intestinalis	0.11	3.88	166486
Ruminococcus gauvreauii	0.12	4.13	438033

(e) Expected relative abundances for the Amos HiLo and Mixed mock communities.

Species	RA (%)	TAXID
Bacteroides uniformis	4.7	820
Blautia sp. NBRC 113351	4.5	2877527
Enterocloster clostridioformis	5.3	1531
Parabacteroides distasonis	4.8	823
Bacillus subtilis	5.2	1423
Streptococcus mutans	6.9	1309
Pseudomonas putida	3.9	303
Lactobacillus delbrueckii	3.6	1584
Escherichia coli	5.6	562
Flavonifractor plautii	3.7	292800
Staphylococcus epidermidis	4.8	1282
Cutibacterium acnes	5.0	1747
Bifidobacterium longum	10.4	216816
Anaerostipes caccae	5.3	105841
Ruminococcus gnavus	5.6	33038
Megasphaera massiliensis	4.8	1232428
Megamonas funiformis	3.7	437897
Collinsella aerofaciens	6.2	74426
Akkermansia muciniphila	6.0	239935

(f) Expected relative abundances for Tourlousse.

Table S1. Expected Relative Abundance tables for all communities.

	bioBakery3	bioBakery4	JAMS	WGSA2
bioBakery4	0.092231	-	-	-
JAMS	**0.040283**	**0.003906**	-	-
WGSA2	**0.040283**	**0.004883**	0.965820	-
Woltka	**0.003255**	**0.003255**	**0.003255**	**0.003906**

(a) Pairwise Wilcoxon test for Aitchison Distance between all pipelines.

	bioBakery3	bioBakery4	JAMS	WGSA2
bioBakery4	0.267242	-	-	-
JAMS	0.093355	0.499571	-	-
WGSA2	0.093355	0.499571	0.499571	-
Woltka	0.858955	0.852109	0.306084	0.093355

(b) Pairwise Wilcoxon test for Sensitivity between all pipelines.

	bioBakery3	bioBakery4	JAMS	WGSA2
Biobakery4	0.777995	-	-	-
JAMS	0.457589	0.777995	-	-
WGSA2	1.000000	0.294922	0.457589	-
Woltka	**0.009766**	**0.014648**	0.104980	0.061849

(c) Pairwise Wilcoxon test for False Positive Relative Abundance between all pipelines.

Table S2. Pairwise Wilcoxon tests for AD, Sens, and FPRA. The FDR (Benjamini-Hochberg procedure) corrected p-values are shown in the table. Bold values indicate FDR-BH < 0.05.

Parameter	Value
amrfind	false
annotations_type	ko
decontaminate	human_mouse
include_ted_files	false
job_desc	–
mag	false
tax_genes	false
tax_scaffold	false
trim_filter	false

Table S3. Table of WGSA2 parameters used.

Species	tp	fp	fn	tn	sens	spec	prec	acc	f1	Threshold
Achromobacter xylosoxidans	3	0	1	1	0.750	1.000	1.000	0.800	0.857	0.0000
Acinetobacter baumannii	3	0	1	1	0.750	1.000	1.000	0.800	0.857	0.0000
Aeromonas hydrophila	4	0	1	0	0.800	-	1.000	0.800	0.889	0.0000
Enterococcus faecalis	4	0	0	1	1.000	1.000	1.000	1.000	1.000	0.0000
Escherichia coli	4	1	0	0	1.000	0.000	0.800	0.800	0.889	0.0000
Klebsiella pneumoniae	3	0	1	1	0.750	1.000	1.000	0.800	0.857	0.0000
Legionella pneumophila	5	0	0	0	1.000	-	1.000	1.000	1.000	0.0000
Listeria monocytogenes	3	0	1	1	0.750	1.000	1.000	0.800	0.857	0.0000
Neisseria meningitidis	3	0	1	1	0.750	1.000	1.000	0.800	0.857	0.0000
Salmonella enterica	3	0	1	1	0.750	1.000	1.000	0.800	0.857	0.0000
Shigella sonnei	0	0	4	1	0.000	1.000	-	0.200	-	0.0000
Staphylococcus aureus	3	0	1	1	0.750	1.000	1.000	0.800	0.857	0.0000
Streptococcus pyogenes	3	0	1	1	0.750	1.000	1.000	0.800	0.857	0.0000
Vibrio furnissii	3	0	1	1	0.750	1.000	1.000	0.800	0.857	0.0000
**Harmonic mean**	-	-	-	-	0.000	0.000	0.981	0.675	0.881	0.0000
**Mean**	-	-	-	-	0.703	0.846	0.984	0.778	0.884	0.0000

(a) Confusion matrix for biobakery3 at threshold 0.0.

Species	tp	fp	fn	tn	sens	spec	prec	acc	f1	Threshold
Achromobacter xylosoxidans	3	0	1	1	0.750	1.000	1.000	0.800	0.857	0.0000
Acinetobacter baumannii	3	0	1	1	0.750	1.000	1.000	0.800	0.857	0.0000
Aeromonas hydrophila	5	0	0	0	1.000	-	1.000	1.000	1.000	0.0000
Enterococcus faecalis	4	0	0	1	1.000	1.000	1.000	1.000	1.000	0.0000
Escherichia coli	4	1	0	0	1.000	0.000	0.800	0.800	0.889	0.0000
Klebsiella pneumoniae	3	0	1	1	0.750	1.000	1.000	0.800	0.857	0.0000
Legionella pneumophila	5	0	0	0	1.000	-	1.000	1.000	1.000	0.0000
Listeria monocytogenes	3	0	1	1	0.750	1.000	1.000	0.800	0.857	0.0000
Neisseria meningitidis	3	0	1	1	0.750	1.000	1.000	0.800	0.857	0.0000
Salmonella enterica	3	1	1	0	0.750	0.000	0.750	0.600	0.750	0.0000
Shigella sonnei	0	0	4	1	0.000	1.000	-	0.200	-	0.0000
Staphylococcus aureus	3	0	1	1	0.750	1.000	1.000	0.800	0.857	0.0000
Streptococcus pyogenes	3	0	1	1	0.750	1.000	1.000	0.800	0.857	0.0000
Vibrio furnissii	4	0	0	1	1.000	1.000	1.000	1.000	1.000	0.0000
**Harmonic mean**	-	-	-	-	0.000	0.000	0.957	0.677	0.889	0.0000
**Mean**	-	-	-	-	0.733	0.769	0.965	0.792	0.895	0.0000

(b) Confusion matrix for biobakery4 at threshold 0.0.

Species	tp	fp	fn	tn	sens	spec	prec	acc	f1	Threshold
Achromobacter xylosoxidans	3	0	1	1	0.750	1.000	1.000	0.800	0.857	0.0000
Acinetobacter baumannii	3	0	1	1	0.750	1.000	1.000	0.800	0.857	0.0000
Aeromonas hydrophila	4	0	1	0	0.800	-	1.000	0.800	0.889	0.0000
Enterococcus faecalis	3	0	1	1	0.750	1.000	1.000	0.800	0.857	0.0000
Escherichia coli	4	1	0	0	1.000	0.000	0.800	0.800	0.889	0.0000
Klebsiella pneumoniae	4	0	0	1	1.000	1.000	1.000	1.000	1.000	0.0000
Legionella pneumophila	5	0	0	0	1.000	-	1.000	1.000	1.000	0.0000
Listeria monocytogenes	3	0	1	1	0.750	1.000	1.000	0.800	0.857	0.0000
Neisseria meningitidis	3	0	1	1	0.750	1.000	1.000	0.800	0.857	0.0000
Salmonella enterica	4	1	0	0	1.000	0.000	0.800	0.800	0.889	0.0000
Shigella sonnei	4	0	0	1	1.000	1.000	1.000	1.000	1.000	0.0000
Staphylococcus aureus	3	0	1	1	0.750	1.000	1.000	0.800	0.857	0.0000
Streptococcus pyogenes	3	0	1	1	0.750	1.000	1.000	0.800	0.857	0.0000
Vibrio furnissii	3	0	1	1	0.750	1.000	1.000	0.800	0.857	0.0000
**Harmonic mean**	-	-	-	-	0.828	0.000	0.966	0.836	0.891	0.0000
**Mean**	-	-	-	-	0.842	0.769	0.971	0.842	0.894	0.0000

(c) Confusion matrix for jams at threshold 0.0.

Species	tp	fp	fn	tn	sens	spec	prec	acc	f1	Threshold
Achromobacter xylosoxidans	4	1	0	0	1.000	0.000	0.800	0.800	0.889	0.0000
Acinetobacter baumannii	4	1	0	0	1.000	0.000	0.800	0.800	0.889	0.0000
Aeromonas hydrophila	5	0	0	0	1.000	-	1.000	1.000	1.000	0.0000
Enterococcus faecalis	4	1	0	0	1.000	0.000	0.800	0.800	0.889	0.0000
Escherichia coli	4	1	0	0	1.000	0.000	0.800	0.800	0.889	0.0000
Klebsiella pneumoniae	4	1	0	0	1.000	0.000	0.800	0.800	0.889	0.0000
Legionella pneumophila	5	0	0	0	1.000	-	1.000	1.000	1.000	0.0000
Listeria monocytogenes	4	1	0	0	1.000	0.000	0.800	0.800	0.889	0.0000
Neisseria meningitidis	4	1	0	0	1.000	0.000	0.800	0.800	0.889	0.0000
Salmonella enterica	4	1	0	0	1.000	0.000	0.800	0.800	0.889	0.0000
Shigella sonnei	4	1	0	0	1.000	0.000	0.800	0.800	0.889	0.0000
Staphylococcus aureus	4	1	0	0	1.000	0.000	0.800	0.800	0.889	0.0000
Streptococcus pyogenes	4	1	0	0	1.000	0.000	0.800	0.800	0.889	0.0000
Vibrio furnissii	4	1	0	0	1.000	0.000	0.800	0.800	0.889	0.0000
**Harmonic mean**	-	-	-	-	1.000	0.000	0.824	0.824	0.903	0.0000
**Mean**	-	-	-	-	1.000	0.000	0.828	0.828	0.905	0.0000

(d) Confusion matrix for wgsa2 at threshold 0.0.

Species	tp	fp	fn	tn	sens	spec	prec	acc	f1	Threshold
Achromobacter xylosoxidans	4	1	0	0	1.000	0.000	0.800	0.800	0.889	0.0000
Acinetobacter baumannii	4	1	0	0	1.000	0.000	0.800	0.800	0.889	0.0000
Aeromonas hydrophila	5	0	0	0	1.000	-	1.000	1.000	1.000	0.0000
Enterococcus faecalis	4	1	0	0	1.000	0.000	0.800	0.800	0.889	0.0000
Escherichia coli	4	1	0	0	1.000	0.000	0.800	0.800	0.889	0.0000
Klebsiella pneumoniae	4	1	0	0	1.000	0.000	0.800	0.800	0.889	0.0000
Legionella pneumophila	5	0	0	0	1.000	-	1.000	1.000	1.000	0.0000
Listeria monocytogenes	4	1	0	0	1.000	0.000	0.800	0.800	0.889	0.0000
Neisseria meningitidis	4	1	0	0	1.000	0.000	0.800	0.800	0.889	0.0000
Salmonella enterica	4	1	0	0	1.000	0.000	0.800	0.800	0.889	0.0000
Shigella sonnei	0	0	4	1	0.000	1.000	-	0.200	-	0.0000
Staphylococcus aureus	4	1	0	0	1.000	0.000	0.800	0.800	0.889	0.0000
Streptococcus pyogenes	4	1	0	0	1.000	0.000	0.800	0.800	0.889	0.0000
Vibrio furnissii	0	0	4	1	0.000	1.000	-	0.200	-	0.0000
**Harmonic mean**	-	-	-	-	0.000	0.000	0.828	0.571	0.906	0.0000
**Mean**	-	-	-	-	0.800	0.154	0.833	0.731	0.907	0.0000

(e) Confusion matrix for woltka at threshold 0.0.

Table S4. Confusion matrices for bioBakery3, bioBakery4, JAMS, WGSA2, and Woltka at 0% filtering threshold. tp = True Positive, fp = False Positive, fn = False Negative, TN = True Negative, sens = Sensitivity, spec = specificity, prec = Precision, acc = Accuracy, f1 = F1 Score

Species	tp	fp	fn	tn	sens	spec	prec	acc	f1	Threshold
Achromobacter xylosoxidans	3	0	1	1	0.750	1.000	1.000	0.800	0.857	0.0001
Acinetobacter baumannii	3	0	1	1	0.750	1.000	1.000	0.800	0.857	0.0001
Aeromonas hydrophila	4	0	1	0	0.800	-	1.000	0.800	0.889	0.0001
Enterococcus faecalis	3	0	1	1	0.750	1.000	1.000	0.800	0.857	0.0001
Escherichia coli	4	1	0	0	1.000	0.000	0.800	0.800	0.889	0.0001
Klebsiella pneumoniae	3	0	1	1	0.750	1.000	1.000	0.800	0.857	0.0001
Legionella pneumophila	5	0	0	0	1.000	-	1.000	1.000	1.000	0.0001
Listeria monocytogenes	3	0	1	1	0.750	1.000	1.000	0.800	0.857	0.0001
Neisseria meningitidis	3	0	1	1	0.750	1.000	1.000	0.800	0.857	0.0001
Salmonella enterica	3	0	1	1	0.750	1.000	1.000	0.800	0.857	0.0001
Shigella sonnei	0	0	4	1	0.000	1.000	-	0.200	-	0.0001
Staphylococcus aureus	3	0	1	1	0.750	1.000	1.000	0.800	0.857	0.0001
Streptococcus pyogenes	3	0	1	1	0.750	1.000	1.000	0.800	0.857	0.0001
Vibrio furnissii	3	0	1	1	0.750	1.000	1.000	0.800	0.857	0.0001
**Harmonic mean**	-	-	-	-	0.000	0.000	0.981	0.667	0.872	0.0001
**Mean**	-	-	-	-	0.687	0.846	0.984	0.764	0.873	0.0001

(a) Confusion matrix for biobakery3 at threshold 0.0001.

Species	tp	fp	fn	tn	sens	spec	prec	acc	f1	Threshold
Achromobacter xylosoxidans	3	0	1	1	0.750	1.000	1.000	0.800	0.857	0.0001
Acinetobacter baumannii	3	0	1	1	0.750	1.000	1.000	0.800	0.857	0.0001
Aeromonas hydrophila	4	0	1	0	0.800	-	1.000	0.800	0.889	0.0001
Enterococcus faecalis	4	0	0	1	1.000	1.000	1.000	1.000	1.000	0.0001
Escherichia coli	4	1	0	0	1.000	0.000	0.800	0.800	0.889	0.0001
Klebsiella pneumoniae	3	0	1	1	0.750	1.000	1.000	0.800	0.857	0.0001
Legionella pneumophila	5	0	0	0	1.000	-	1.000	1.000	1.000	0.0001
Listeria monocytogenes	3	0	1	1	0.750	1.000	1.000	0.800	0.857	0.0001
Neisseria meningitidis	3	0	1	1	0.750	1.000	1.000	0.800	0.857	0.0001
Salmonella enterica	3	1	1	0	0.750	0.000	0.750	0.600	0.750	0.0001
Shigella sonnei	0	0	4	1	0.000	1.000	-	0.200	-	0.0001
Staphylococcus aureus	3	0	1	1	0.750	1.000	1.000	0.800	0.857	0.0001
Streptococcus pyogenes	3	0	1	1	0.750	1.000	1.000	0.800	0.857	0.0001
Vibrio furnissii	4	0	0	1	1.000	1.000	1.000	1.000	1.000	0.0001
**Harmonic mean**	-	-	-	-	0.000	0.000	0.957	0.669	0.881	0.0001
**Mean**	-	-	-	-	0.720	0.769	0.965	0.778	0.886	0.0001

(b) Confusion matrix for biobakery4 at threshold 0.0001.

Species	tp	fp	fn	tn	sens	spec	prec	acc	f1	Threshold
Achromobacter xylosoxidans	3	0	1	1	0.750	1.000	1.000	0.800	0.857	0.0001
Acinetobacter baumannii	3	0	1	1	0.750	1.000	1.000	0.800	0.857	0.0001
Aeromonas hydrophila	0	0	5	0	0.000	-	-	0.000	-	0.0001
Enterococcus faecalis	3	0	1	1	0.750	1.000	1.000	0.800	0.857	0.0001
Escherichia coli	4	1	0	0	1.000	0.000	0.800	0.800	0.889	0.0001
Klebsiella pneumoniae	3	0	1	1	0.750	1.000	1.000	0.800	0.857	0.0001
Legionella pneumophila	4	0	1	0	0.800	-	1.000	0.800	0.889	0.0001
Listeria monocytogenes	3	0	1	1	0.750	1.000	1.000	0.800	0.857	0.0001
Neisseria meningitidis	3	0	1	1	0.750	1.000	1.000	0.800	0.857	0.0001
Salmonella enterica	4	1	0	0	1.000	0.000	0.800	0.800	0.889	0.0001
Shigella sonnei	3	0	1	1	0.750	1.000	1.000	0.800	0.857	0.0001
Staphylococcus aureus	3	0	1	1	0.750	1.000	1.000	0.800	0.857	0.0001
Streptococcus pyogenes	3	0	1	1	0.750	1.000	1.000	0.800	0.857	0.0001
Vibrio furnissii	3	0	1	1	0.750	1.000	1.000	0.800	0.857	0.0001
**Harmonic mean**	-	-	-	-	0.000	0.000	0.963	0.000	0.864	0.0001
**Mean**	-	-	-	-	0.687	0.769	0.969	0.693	0.864	0.0001

(c) Confusion matrix for jams at threshold 0.0001.

Species	tp	fp	fn	tn	sens	spec	prec	acc	f1	Threshold
Achromobacter xylosoxidans	4	0	0	1	1.000	1.000	1.000	1.000	1.000	0.0001
Acinetobacter baumannii	4	0	0	1	1.000	1.000	1.000	1.000	1.000	0.0001
Aeromonas hydrophila	5	0	0	0	1.000	-	1.000	1.000	1.000	0.0001
Enterococcus faecalis	4	0	0	1	1.000	1.000	1.000	1.000	1.000	0.0001
Escherichia coli	4	1	0	0	1.000	0.000	0.800	0.800	0.889	0.0001
Klebsiella pneumoniae	4	0	0	1	1.000	1.000	1.000	1.000	1.000	0.0001
Legionella pneumophila	5	0	0	0	1.000	-	1.000	1.000	1.000	0.0001
Listeria monocytogenes	4	0	0	1	1.000	1.000	1.000	1.000	1.000	0.0001
Neisseria meningitidis	4	0	0	1	1.000	1.000	1.000	1.000	1.000	0.0001
Salmonella enterica	4	1	0	0	1.000	0.000	0.800	0.800	0.889	0.0001
Shigella sonnei	0	0	4	1	0.000	1.000	-	0.200	-	0.0001
Staphylococcus aureus	3	0	1	1	0.750	1.000	1.000	0.800	0.857	0.0001
Streptococcus pyogenes	4	0	0	1	1.000	1.000	1.000	1.000	1.000	0.0001
Vibrio furnissii	4	0	0	1	1.000	1.000	1.000	1.000	1.000	0.0001
**Harmonic mean**	-	-	-	-	0.000	0.000	0.963	0.747	0.969	0.0001
**Mean**	-	-	-	-	0.850	0.769	0.969	0.890	0.972	0.0001

(d) Confusion matrix for wgsa2 at threshold 0.0001.

Species	tp	fp	fn	tn	sens	spec	prec	acc	f1	Threshold
Achromobacter xylosoxidans	4	0	0	1	1.000	1.000	1.000	1.000	1.000	0.0001
Acinetobacter baumannii	4	0	0	1	1.000	1.000	1.000	1.000	1.000	0.0001
Aeromonas hydrophila	5	0	0	0	1.000	-	1.000	1.000	1.000	0.0001
Enterococcus faecalis	4	0	0	1	1.000	1.000	1.000	1.000	1.000	0.0001
Escherichia coli	4	1	0	0	1.000	0.000	0.800	0.800	0.889	0.0001
Klebsiella pneumoniae	4	1	0	0	1.000	0.000	0.800	0.800	0.889	0.0001
Legionella pneumophila	5	0	0	0	1.000	-	1.000	1.000	1.000	0.0001
Listeria monocytogenes	4	0	0	1	1.000	1.000	1.000	1.000	1.000	0.0001
Neisseria meningitidis	4	0	0	1	1.000	1.000	1.000	1.000	1.000	0.0001
Salmonella enterica	4	1	0	0	1.000	0.000	0.800	0.800	0.889	0.0001
Shigella sonnei	0	0	4	1	0.000	1.000	-	0.200	-	0.0001
Staphylococcus aureus	4	0	0	1	1.000	1.000	1.000	1.000	1.000	0.0001
Streptococcus pyogenes	3	0	1	1	0.750	1.000	1.000	0.800	0.857	0.0001
Vibrio furnissii	0	0	4	1	0.000	1.000	-	0.200	-	0.0001
**Harmonic mean**	-	-	-	-	0.000	0.000	0.941	0.609	0.957	0.0001
**Mean**	-	-	-	-	0.783	0.692	0.949	0.814	0.960	0.0001

(e) Confusion matrix for woltka at threshold 0.0001.

Table S5. Confusion matrices for bioBakery3, bioBakery4, JAMS, WGSA2, and Woltka at 0.1% filtering threshold. tp = True Positive, fp = False Positive, fn = False Negative, TN = True Negative, sens = Sensitivity, spec = specificity, prec = Precision, acc = Accuracy, f1 = F1 Score

	bioBakery3	bioBakery4	JAMS	WGSA2	Woltka
Time per CPU (HH:MM:SS)	00:07:23	00:01:09	00:01:33	00:01:43	00:26:45
Relative Time Per CPU (%)	534.4	-	33.0	47.8	2194.5

Table S6. Relative Time per CPU for all NIST samples. If multiple samples were run in parallel, the average was taken. The Woltka time is the sum of both the alignment and classification steps for each sample.

	**Number False Positive**											
	**Sample Type**	**n**	**bioBakery3**		**bioBakery4**		**JAMS**		**WGSA2**		**Woltka**	
			Mean	Stdev	Mean	Stdev	Mean	Stdev	Mean	Stdev	Mean	Stdev
One-to-One	BMock12	1	3		2		31		72		98	
	CamiSim S1	1	3		3		13		21		55	
	CamiSim S2	1	1		1		4		24		75	
	NIST EG	1	5		0		11		14		226	
	NIST MIX-A	1	4		0		3		14		128	
	NIST MIX-B	1	5		1		4		17		180	
	NIST MIX-C	1	3		1		7		16		136	
	NIST MIX-D	1	1		1		2		6		120	
Replicates	Amos HiLo	5	0.20	0.45	0.00	0.00	8.20	0.84	21.80	1.64	117.20	1.92
	Amos Mixed	5	1.00	0.00	0.00	0.00	27.20	1.30	46.20	0.84	212.40	3.91
	Tourlousse	6	7.83	0.41	1.00	0.00	14.17	2.14	35.17	0.41	239.00	3.29
**Average**			3.09	2.28	0.91	0.94	11.32	9.71	26.11	18.77	144.24	61.75

Table S7. Table of the Number of False Positive species. A perfect score is 0.

	**Unclassified**												
	**Sample Type**	**n**	**Biobakery3**		**Biobakery4**		**JAMS**		**WGSA2**		**Woltka**		**Average**
			Mean	Stdev	Mean	Stdev	Mean	Stdev	Mean	Stdev	Mean	Stdev	
One-to-One	BMock12	1	-	-	-	-	1.32	0.00	28.53	0.00	-	-	5.97
	CamiSim S1	1	-	-	-	-	2.25	0.00	0.88	0.00	-	-	0.63
	CamiSim S2	1	-	-	-	-	0.85	0.00	0.08	0.00	-	-	0.18
	NIST EG	1	-	-	-	-	15.70	0.00	0.59	0.00	-	-	3.26
	NIST MIX-A	1	-	-	-	-	3.95	0.00	0.24	0.00	-	-	0.84
	NIST MIX-B	1	-	-	-	-	4.85	0.00	0.25	0.00	-	-	1.02
	NIST MIX-C	1	-	-	-	-	6.42	0.00	0.22	0.00	-	-	1.33
	NIST MIX-D	1	-	-	-	-	2.86	0.00	0.20	0.00	-	-	0.61
Replicates	Amos HiLo	5	-	-	-	-	3.08	0.08	3.34	0.11	-	-	1.28
	Amos Mixed	5	-	-	-	-	7.63	1.55	9.59	0.07	-	-	3.44
	Tourlousse	6	-	-	-	-	3.23	0.30	12.37	0.13	-	-	3.12
**Average**			0.00	0.00	0.00	0.00	4.74	4.17	5.12	8.85	0.00	0.00	0.00

Table S8. Table of Unclassified metrics. The values shown are percentages. A perfect score is 0%. Neither bioBakery nor Woltka give the number of unclassified by default.

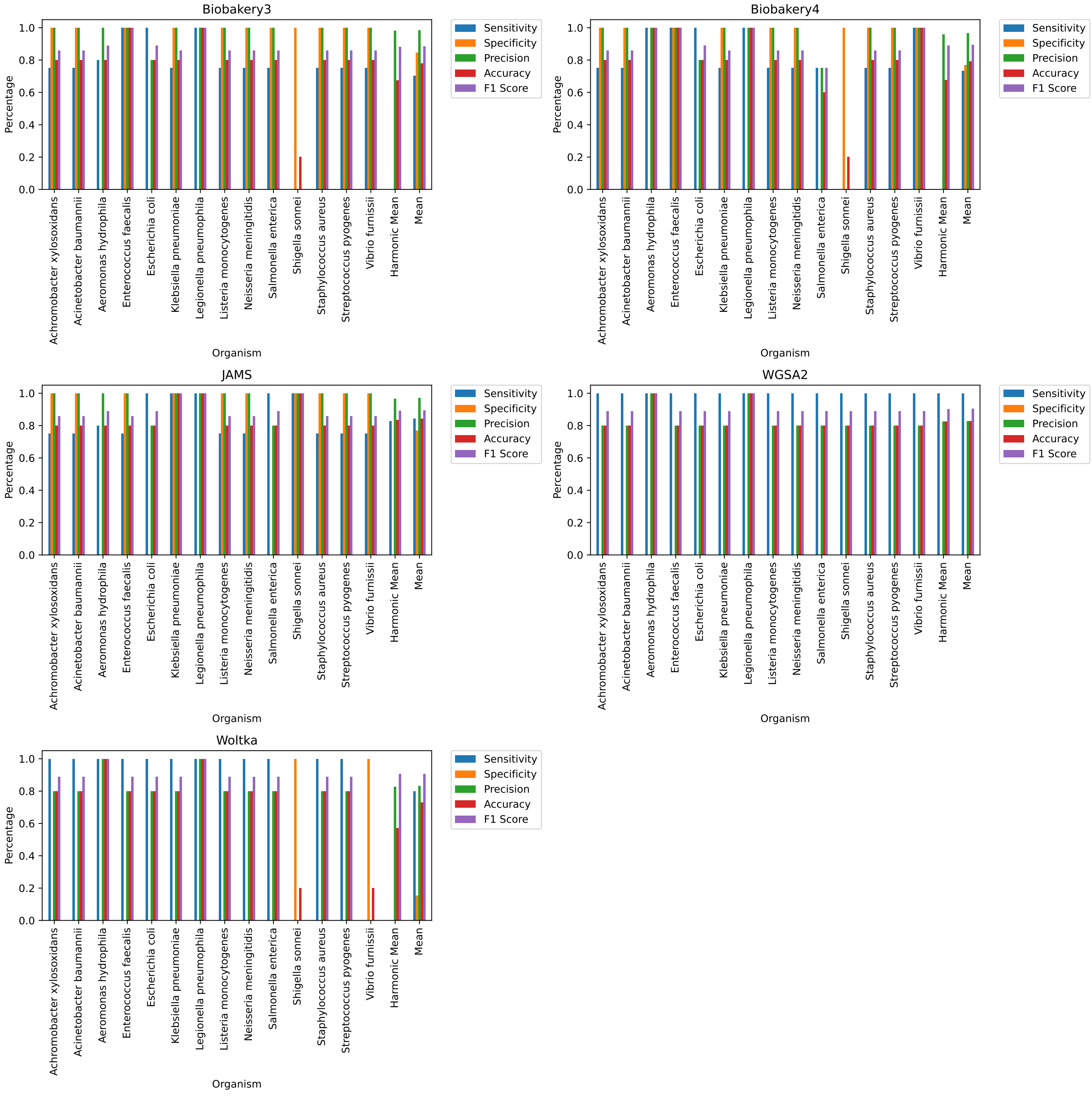

Figure S1. Bar Plot graphical representation of confusion matrices shown in Supplemental Table S4.

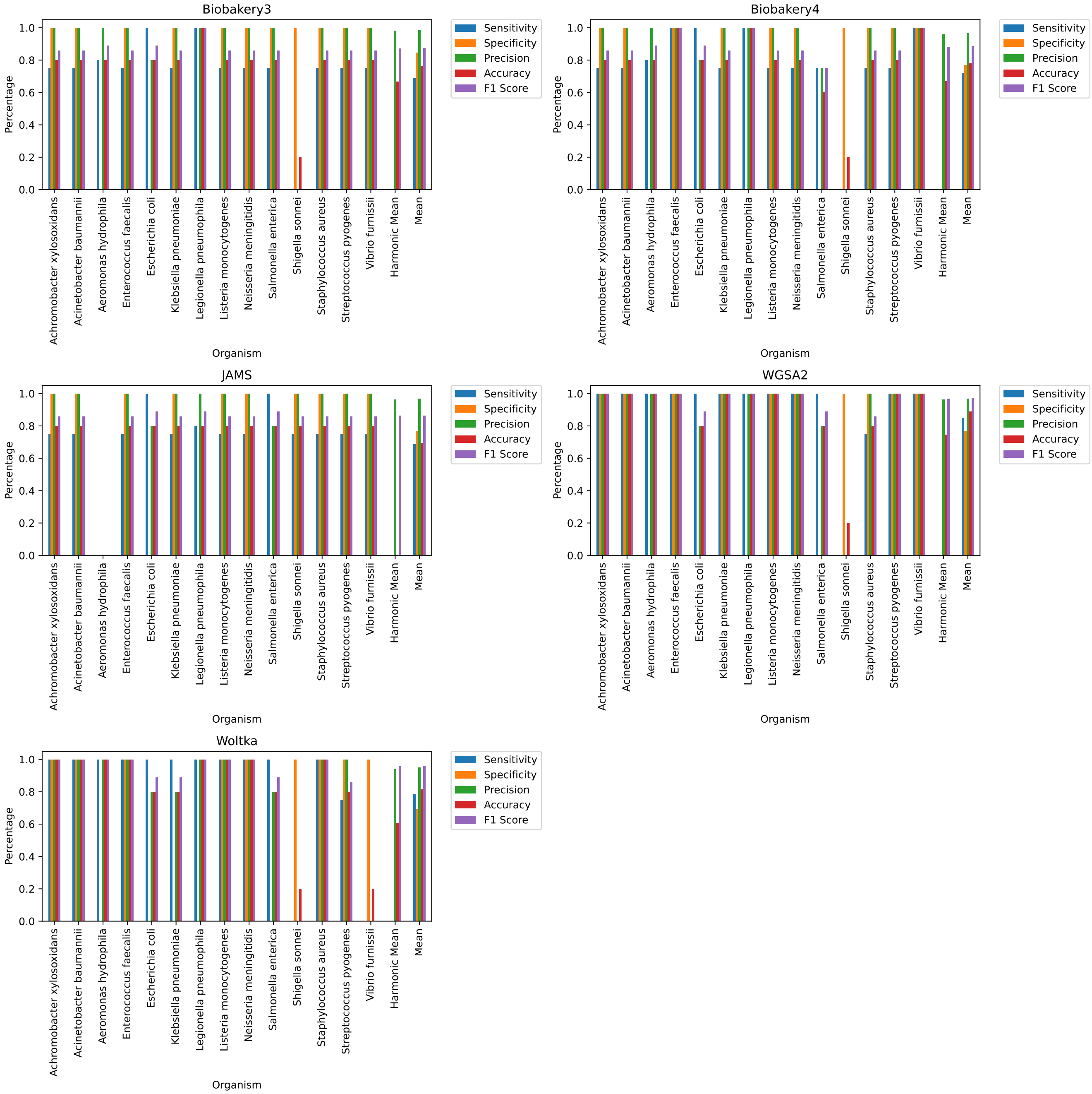

Figure S2. Bar Plot graphical representation of confusion matrices shown in Supplemental Table S5.
